# A shared alarmone–GTP switch controls persister formation in bacteria

**DOI:** 10.1038/s41564-025-02015-6

**Published:** 2025-05-15

**Authors:** Danny K. Fung, Jessica T. Barra, Jin Yang, Jeremy W. Schroeder, Fukang She, Megan Young, David Ying, David M. Stevenson, Daniel Amador-Noguez, Jue D. Wang

**Affiliations:** Department of Bacteriology, University of Wisconsin, Madison, WI USA

**Keywords:** Bacterial genetics, Bacteriology

## Abstract

Persisters are phenotypically switched bacteria that survive antibiotic exposure despite being genetically susceptible. Three pathways to persistence—triggered, spontaneous and antibiotic-induced—have been described, but the underlying molecular mechanisms are poorly understood. Here, we used antibiotic time–kill assays as well as single-cell approaches to show that all of the pathways depend on a common switch involving the alarmone guanosine tetra/penta-phosphate ((p)ppGpp) in *Bacillus subtilis*, each stemming from different alarmone synthetase(s). The accumulation of (p)ppGpp promotes persistence through depletion of intracellular GTP. We developed a fluorescent GTP reporter to visualize rare events of persister formation in wild-type bacteria, revealing a rapid switch from growth to dormancy in single cells as their GTP levels drop beneath a threshold. While a decrease in GTP in the bulk population slows growth and promotes antibiotic tolerance, (p)ppGpp drives persistence by driving rapid, switch-like decreases in GTP levels beneath the persister threshold in single cells. Persistence through alarmone–GTP antagonism is probably a widespread mechanism to survive antibiotics in *B. subtilis* and potentially beyond.

## Abstract (web summary)

Alarmone synthesis depletes GTP levels leading to a GTP-dependent switch that controls triggered, spontaneous and antibiotic-induced persister formation in *Bacillus subtilis*.

## Main

Antibiotic persisters are individual bacterial cells that survive prolonged bactericidal antibiotic treatment despite being genetically sensitive to antibiotics^1–3^. Joseph Bigger originally proposed that persisters can be generated before or after treatment with antibiotics^2^. Later, persisters were categorized by their pathways of generation—starvation triggered, spontaneously generated and antibiotic induced^1^—yet their molecular mechanisms remain unclear. Although persistence can be triggered by nutrient deprivations^4^ through the intracellular accumulation of the alarmone (p)ppGpp^5–9^, it is also argued that slow growth or a reduction in ATP levels, rather than (p)ppGpp, is responsible for triggering persistence^10,11^. In addition to starvation, persisters can form spontaneously^3^. However, spontaneous persisters exist in natural populations at a low frequency, making their identification challenging and making it difficult to establish the mechanisms of their formation. While toxin–antitoxin (TA) modules are strongly implicated in persistence^3,5,8,12,13^, their roles in spontaneous antibiotic persistence of wild-type bacteria remain unclear^14^. Finally, persisters were observed both before and after treatment with antibiotics^15^, and bacteriostatic antibiotics are known to induce persisters^16^. However, it is difficult to differentiate antibiotic-induced persistence from antibiotic tolerance^1^, spontaneous persistence, transcriptional response to specific drugs or by-products of cellular damage from drug treatment^1^. The mechanisms of antibiotic-induced persistence remain poorly understood. Here we directly visualize the formation of starvation-triggered, spontaneous and antibiotic-induced persisters in the Gram-positive bacterium *B. subtilis* belonging to the phylum Bacillota and reveal that multiple cellular mechanisms of persistence can converge on a common pathway of alarmone–GTP antagonism.

## Results

### (p)ppGpp accumulation drives starvation-triggered persistence

**Fig. 1:**
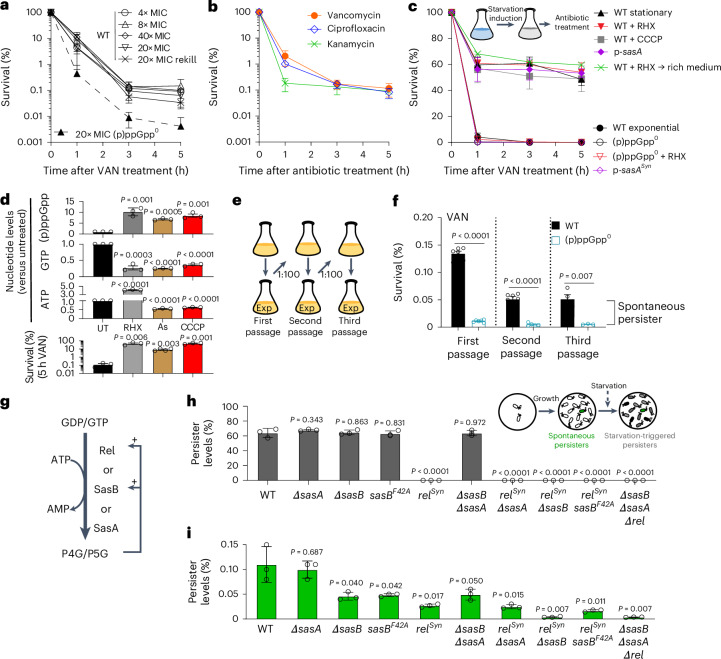
(p)ppGpp mediates starvation-triggered and spontaneous persistence. **a**, Survival curves of *B. subtilis* wild-type (WT) and (p)ppGpp^0^ after treatment with lethal concentrations of vancomycin (VAN). Rekill indicates that cells that survived 20× MIC vancomycin treatment were regrown and treated with vancomycin again. **b**, Survival curves of prophage-cured WT *B. subtilis* treated with 20× MIC vancomycin, 20× MIC ciprofloxacin or 8× MIC kanamycin. **c**, Starvation-triggered persistence of WT *B. subtilis* depends on (p)ppGpp. Biphasic kill curve assays of vancomycin treatment were performed after treatment with several starvation-inducing or (p)ppGpp-inducing conditions. Stationary, stationary phase; RHX, pretreated with the amino acid starvation inducer arginine hydroxamate; CCCP, pretreated with ATP synthesis inhibitor CCCP; p-*sasA* or p-*sasA*^*Syn*^, (p)ppGpp^0^ strains overexpressing the (p)ppGpp synthetase SasA or its synthetase-dead variant SasA^Syn^ (*sasA*^*D87G*^). Plots shown on a log scale are provided in Extended Data Fig. 2. **d**, The nucleotide levels in cells pretreated with arginine hydroxamate or ATP synthesis inhibitor CCCP or arsenate (As) for 30 min (top). UT, untreated. The nucleotide levels were measured using thin-layer chromatography (TLC) and normalized to their levels before induction. Bottom, persistence of the pretreated cells to vancomycin treatment. **e**,**f**, Schematic (**e**) and quantification (**f**) of the survival of exponential phase (Exp), serial-passaged populations after vancomycin treatment for 5 h. Persisters that remained through the passage were defined as spontaneous persisters. **g**, In *B. subtilis* and many Gram-positive bacteria, (p)ppGpp is synthesized by three (p)ppGpp synthetases. Rel is the bifunctional (p)ppGpp synthetase and hydrolase, whereas SasB (also known as SAS1, RelQ or YjbM) and SasA (also known as SAS2, RelP or YwaC) are monofunctional synthetases. **h**, Persister levels in starvation-induced (arginine hydroxamate; black) WT and (p)ppGpp mutants. **i**, The levels of spontaneous persisters (green, second passage) in WT and (p)ppGpp mutants. *rel*^*Syn*^ (*rel*^*D264G*^) is a synthetase-inactive variant of Rel that retains its hydrolase activity essential for viability in the presence of *sasB* and *sasA*. *sasB*^*F42A*^ encodes an allosteric pppGpp-binding-site mutant of SasB. For **a**–**d**, **f**, **h** and **i**, data are mean ± s.d. from three or more biological replicates. For **d**, **f**, **h** and **i**, *P* values were calculated using unpaired two-tailed *t*-tests.

**Extended Data Fig. 1:**
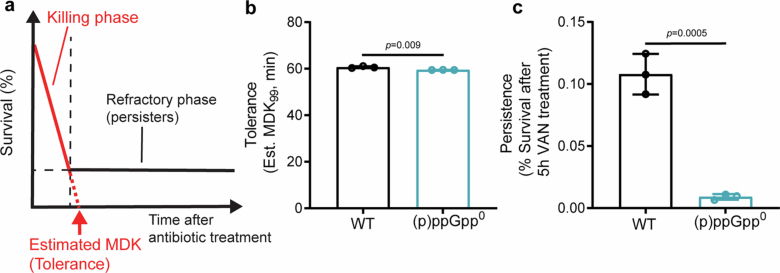
(p)ppGpp has a strong effect on persistence (and a modest effect on tolerance). (**a**) Measuring tolerance from kill curve. The biphasic killing curve is composed of two phases: an initial killing phase (red) where the non-persister population dies rapidly upon antibiotic treatment, followed by an antibiotic-refractory phase (black) where persisters survive prolonged treatment. To measure tolerance of the bulk population, we subtracted the persister fraction from the population and estimated its MDK_99_ (minimum duration of killing for 99% cells) from the killing phase. (**b-c**) Tolerance (**b**) and persistence (**c**) of exponentially growing WT or (p)ppGpp^0^ mutant after 5 h vancomycin treatment. Values represent mean and error bars represent s.d. from three biological replicates. *p* values were derived from unpaired two-tailed t-test.

To quantify persistence in *B. subtilis*, we began by treating cells with the cell-wall-targeting antibiotic vancomycin at concentrations of 20 times higher than its minimal inhibitory concentration (MIC) (Fig. 1a and Supplementary Fig 1). The results revealed biphasic killing kinetics, with a rapid elimination of the majority of cells followed by the prolonged survival of a small subpopulation. This surviving fraction (approximately 0.1%) remained consistent across varying drug concentrations and, after regrowth, became resensitized to antibiotics (Fig. 1a), confirming that these are phenotypically switched persisters rather than genetically altered resisters^1,2,17^. We also tested the effect of prophages on persistence, given that persistence against the DNA-damaging antibiotic ciprofloxacin has been shown to be complicated by prophage activation in *Escherichia coli*^14^. We confirmed that, similar to *E. coli*, persistence against ciprofloxacin is weakened by prophages (Supplementary Fig. 2), but persistence against vancomycin remains unaffected regardless of the presence or absence of resident prophages SPβ and PBSX within the genome. Furthermore, the levels of persisters in prophage-cured cells were consistent across different classes of antibiotics, including vancomycin, ciprofloxacin and kanamycin, that target translation (Fig. 1b). This suggests that the persisters originate from a common subpopulation that is refractory to multiple antibiotics^1^.

(p)ppGpp is a starvation-induced alarmone that is strongly implicated in antibiotic survival^9^. We therefore examined the effect of (p)ppGpp on antibiotic resistance, tolerance and persistence using mutant *B. subtilis* lacking all three (p)ppGpp synthetases (hereafter, (p)ppGpp^0^)^18,19^. Compared with the wild-type strain, the (p)ppGpp^0^ strain exhibited similar resistance to a variety of antibiotics (MIC assay; Supplementary Fig. 1), a modestly reduced antibiotic tolerance (approximately 5% reduction in MDK_99_, where MDK_99_ is the minimum duration of killing for 99% of the population; Extended Data Fig. 1a,b) and strongly reduced (around tenfold) antibiotic persistence (Fig. 1a and Extended Data Fig. 1c). These results indicate that (p)ppGpp predominantly promotes antibiotic persistence.

**Extended Data Fig. 2:**
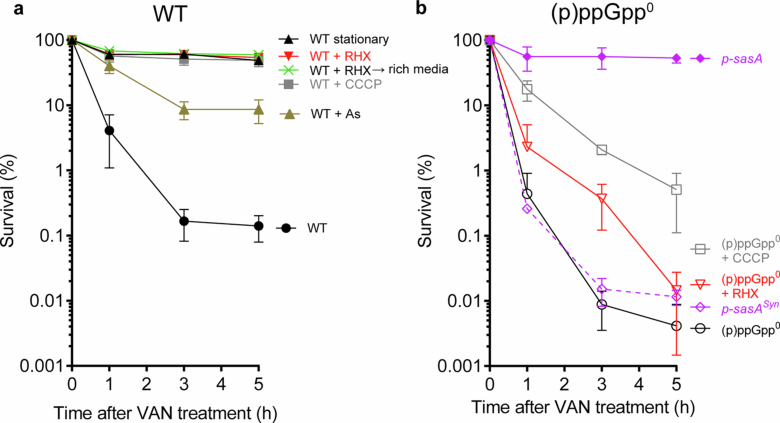
Replot of Fig. 1c in log scale. (**a**) Vancomycin survival curves of WT cells after starvation or (p)ppGpp induction. stationary: stationary phase, RHX: pre-treated with amino acid starvation inducer arginine hydroxamate, +CCCP: pre-treated with ATP synthesis inhibitor CCCP, +As: pre-treated with ATP synthesis inhibitor arsenate. Values represent mean and error bars represent s.d. from three biological replicates. (**b**) Vancomycin survival curves of (p)ppGpp^0^ after starvation or (p)ppGpp induction. RHX: pre-treated with amino acid starvation inducer arginine hydroxamate, +CCCP: pre-treated with ATP synthesis inhibitor CCCP, p-*sasA* or p-*sasA*^*Syn*^: (p)ppGpp^0^ strains overexpressing the (p)ppGpp synthetase SasA or its synthetase-dead variant SasA^Syn^ (SasA^D87G^). Values represent mean and error bars represent s.d. from three biological replicates. See also Supplemental Data Table 5 for a summary of resistance, tolerance and persistence in starvation-induced wild type and (p)ppGpp^0^ mutant.

Antibiotic persistence can be triggered by starvation or occur spontaneously^1^. To investigate the role of (p)ppGpp in starvation-triggered persistence, we starved cells using several methods: allowing growth into stationary phase, inducing amino acid starvation, or depleting ATP with arsenate^10^ or CCCP^20^. We then performed kill curves with vancomycin under these conditions or after starvation was removed by back-diluting in rich medium. The biphasic kill curves (Fig. 1c and Extended Data Fig. 2a) showed a high level of triggered persisters (around 500-fold increase from 0.1% to about 50%) after all starvation treatments and even after starvation removal, consistent with the established definition of triggered persistence^1^.

Notably, all starvation conditions also induced (p)ppGpp accumulation. We observed (p)ppGpp buildup not only after amino acid starvation but also during ATP depletion (Fig. 1d and Supplementary Fig. 3). Furthermore, starvation-triggered persistence was abolished in the (p)ppGpp^0^ mutant, indicating that (p)ppGpp induction is necessary for triggered persistence (Extended Data Fig. 2b and Supplementary Table 5). Finally, we found that, even in the absence of starvation, gratuitous induction of (p)ppGpp by overexpressing its synthetase^21^ was sufficient to produce high levels of persisters (Fig. 1c; *p-sasA*). These findings demonstrate that (p)ppGpp accumulation is both necessary and sufficient to trigger persistence.

### (p)ppGpp mediates spontaneous persistence through positive feedback

We next investigated the occurrence of spontaneous persistence. To differentiate spontaneous persisters from those triggered by starvation, we performed a classical serial passage experiment^1,3^ (Fig. 1e). In this method, cells are repeatedly diluted into fresh medium during exponential growth, which removes starvation-triggered persisters from the lag-phase culture while leaving a consistent, basal fraction of spontaneously generated persisters. This approach enabled us to identify a rare population of spontaneous persisters (around 0.05%) after the starvation-triggered persisters have been eliminated (Fig. 1f). Notably, the spontaneous persisters were absent in the (p)ppGpp^0^ mutant, indicating that (p)ppGpp has a key role in both spontaneous and starvation-triggered persistence.

In *B. subtilis*, (p)ppGpp is synthesized by three enzymes: Rel, SasA and SasB (Fig. 1g). To assess their roles in persister formation, we analysed single, double and triple mutants of these synthetases (Fig. 1h,i, Supplementary Figs. 4 and 5 and Supplementary Tables 6 and 7). Our findings show that only the starvation-responsive synthetase, Rel, is essential for starvation-triggered persistence (Fig. 1h). By contrast, spontaneous persistence is controlled by both Rel and SasB, as mutations in either *rel* or *sasB* significantly reduce spontaneous persister levels and, in combination, they completely abolish this phenotype (Fig. 1i). Rel can be activated by pppGpp (Supplementary Fig. 6), aligning with recent findings^22^. SasB, a tetrameric enzyme, enables allosteric activation of (p)ppGpp synthesis by pppGpp, with a Hill coefficient of around 3.0 (ref. ^23^). A mutation that disrupts the allosteric pppGpp-binding site in SasB (*sasB*^*F42A*^) leads to a similar decrease in spontaneous persistence as observed in the Δ*sasB* mutant (Fig. 1i). These results suggest that self-amplification of (p)ppGpp synthesis is crucial for spontaneous persister formation.

### GTP decrease downstream of (p)ppGpp mediates persistence

**Fig. 2:**
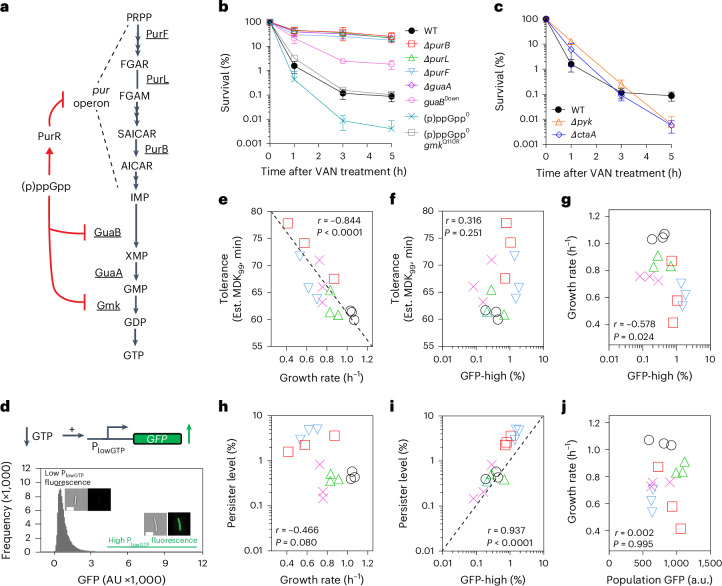
(p)ppGpp mediates persistence by reducing GTP and is independent of tolerance. **a**, (p)ppGpp robustly inhibits de novo GTP biosynthesis by inhibiting the guanylate kinase Gmk and the IMP dehydrogenase GuaB, as well as inhibiting expression of the *pur* operon through the PurR repressor. (p)ppGpp also inhibits GTP synthesis by purine salvage, which is not illustrated here. **b**,**c**, Vancomycin-survival curves of WT, purine-biosynthesis mutants ∆*purB*, ∆*purL* and ∆*purF*, the GTP-synthesis mutant ∆*guaA* and the *guaB*-repressed mutant (*guaB*^*Down*^) (**b**); the slow-growth mutants ∆*pyk* (pyruvate kinase) and ∆*ctaA* (haem synthase) (**c**); and the (p)ppGpp^0^ and (p)ppGpp^0^ *gmk*^*Q110R*^ strains (**b**). Data are mean ± s.d. from three biological replicates. **d**, Measurement of low-GTP cells in the bacterial population. The P_lowGTP_ reporter is activated when cellular GTP levels are low. Flow cytometry analysis of the WT population containing the reporter (around 1.5 × 10^6^ cells). A representative image of cells with low or high P_lowGTP_ fluorescence is shown. Scale bar, 5 µm. **e**–**j**, The correlation between the growth rate (**e**,**g**,**h**,**j**), tolerance (**e**,**f**), persistence (**h**,**i**), population-averaged P_lowGTP_ signals (**j**) and frequency of low-GTP cells (**f**,**g**,**i**; high P_lowGTP_ fluorescence cells) in WT cells under different growth conditions. WT cells containing the P_lowGTP_ reporter were grown in minimal medium with different carbon sources. Circles denote glucose; upright triangles denote pyruvate; crosses denote glycerol; inverted triangles denote succinate; and squares denote glutamate. Populations were measured for their growth rate, P_lowGTP_ fluorescence and levels of high P_lowGTP_ fluorescence cells using flow cytometry (around 1.5 × 10^6^ cells), and their tolerance and persistence were determined on the basis of vancomycin-survival curves as shown in Extended Data Fig. 5. Each value represents a single replicate. Three replicates for each condition are shown. *r* represents the Pearson correlation coefficient. *P* values were calculated using two-tailed Pearson correlation tests. Est., estimated.

We next searched for the downstream effector of (p)ppGpp that mediates antibiotic persistence. (p)ppGpp is a bacterial alarmone with pleiotropic functions including upregulating competence and sporulation^24^. We found that disruption of competence, sporulation or three known TA systems in *B. subtilis* has little impact on persistence (Supplementary Fig. 7). (p)ppGpp also antagonizes the essential nucleotide GTP in *B. subtilis* and downregulates macromolecular biosynthesis^18^. (p)ppGpp production consumes GTP, and (p)ppGpp directly inhibits multiple enzymes along the pathway of GTP production^18,25–27^ (Fig. 2a). Moreover, (p)ppGpp binds to the purine transcription regulator PurR to inhibit transcription of the *pur* operon, encoding de novo purine biosynthesis genes upstream of GTP synthesis^28^. Furthermore, (p)ppGpp competes with GTP to regulate macromolecule synthesis enzymes, including the DNA replication enzyme primase^29,30^, and GTPases for ribosome biogenesis, assembly and protein translation^31–34^. Under starvation conditions that trigger persisters, GTP levels were significantly depleted, whereas ATP levels varied (Fig. 1d), suggesting that GTP depletion may be a common effector leading to persistence.

**Extended Data Fig. 3:**
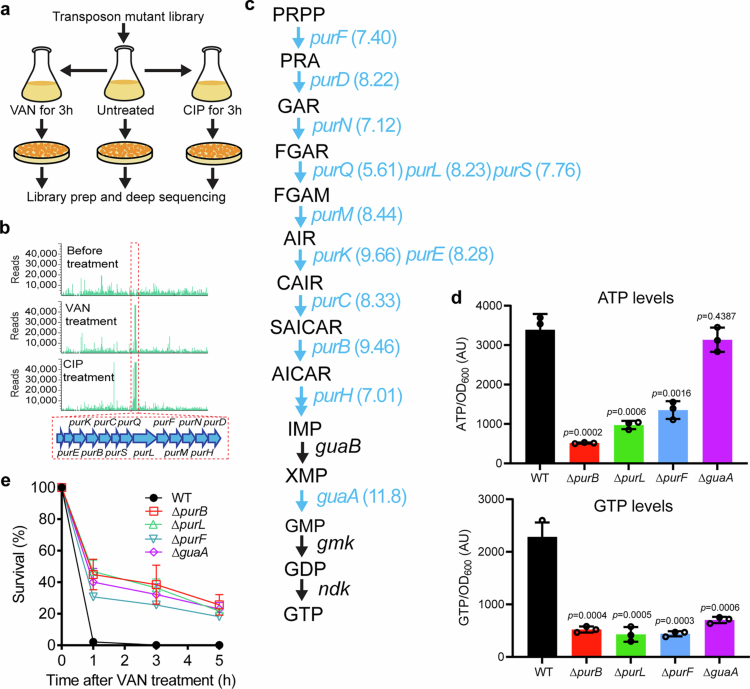
Genome-wide Tn-Seq screen identified strong enrichment of purine biosynthesis gene disruptions after antibiotic treatment. (**a**) Schematic of Tn-Seq experiment. Transposon mutant library was grown to log phase followed by treatment with Vancomycin or Ciprofloxacin. Populations before and after treatment were sampled for sequencing library preparation and deep sequencing. In this assay, only insertions in non-essential or conditionally essential genes (due to availability of casamino acids) will be selected. (**b**) Genome-wide insertion profile from the experiment in (**a**), red box indicates enrichment of the *pur* operon disruptions after antibiotic treatment. (**c**) Pathway of *de novo* purine and GTP biosynthesis. Enriched gene disruptions in the *pur* operon and *guaA* from the Tn-Seq library screen were highlighted in cyan. Numbers in parentheses indicate relative enrichment of disruptions in log_2_ (vs before Vancomycin treatment). Overall, the insertions before the IMP step would all lower GTP and ATP synthesis, while *guaA* insertion would lower GTP but not ATP synthesis. *guaB* and *gmk* have been reported as essential genes and were not present in the library. (**d**) ATP and GTP levels of WT, ∆*purB*, ∆*purL*, ∆*purF*, and ∆*guaA*. Nucleotide levels were measured by thin layer chromatography (TLC) and normalized to OD_600_. Values represent mean and error bars indicate s.d. from three biological replicates. *p* values were derived from unpaired two-tailed t-test between WT and mutant pairs. (**e**) Vancomycin survival curves of WT, ∆*purB*, ∆*purL*, ∆*purF*, and ∆*guaA*. Values represent mean and error bars represent s.d. from three biological replicates. Importantly, *guaA* deletion which only lower GTP but not ATP showed the same increase in persistence as the *pur* mutants. This implies that GTP depletion is critical to persistence.

**Extended Data Fig. 4:**
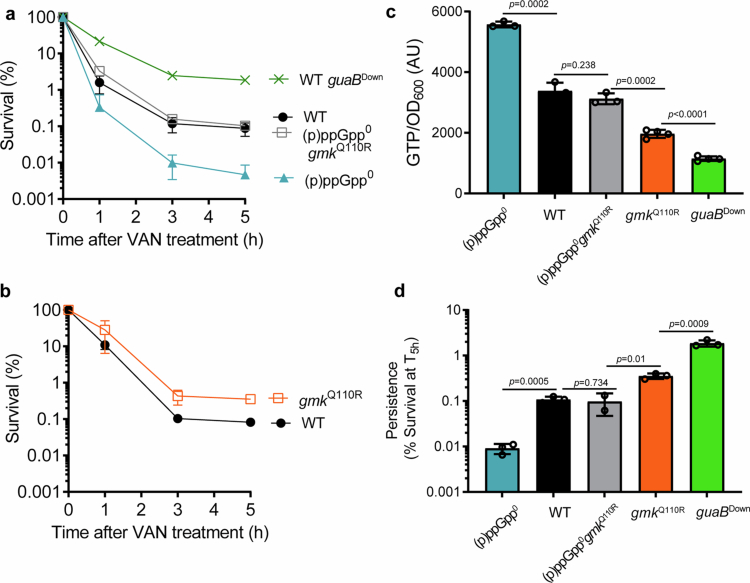
Genetically lowering GTP can restore persistence in the (p)ppGpp^0^ mutant or increase persistence in wildtype. (**a-b**) Vancomycin survival curves of WT, *gmk*^*Q110R*^,(p)ppGpp^0^, (p)ppGpp^0^ *gmk*^*Q110R*^, and *guaB* repressed mutant (*guaB*^Down^). WT and *gmk*^*Q110R*^ kill curve in (**b**) was performed at 30⁰C to minimize the generation of suppressor mutant in *gmk*^*Q110R*^ in (p)ppGpp^+^ background. Values represent mean and error bars represent s.d. from three biological replicates. (**c**-**d**) GTP levels (**c**) and persister levels (**d**) of WT, *gmk*^*Q110R*^,(p)ppGpp^0^, (p)ppGpp^0^ *gmk*^*Q110R*^, and *guaB* repressed mutant (*guaB*^Down^). Nucleotide levels were measured by thin layer chromatography (TLC) and normalized to OD_600_. Values represent mean and error bars represent s.d. from three biological replicates. *p* values were derived from unpaired two-tailed *t*-test.

To test whether GTP depletion mediates antibiotic persistence even in the absence of (p)ppGpp induction, we used our previously published strain with inducible downregulated expression of the guanine nucleotide synthesis gene *guaB*^35^ (*guaB*^*Down*^), lowering GTP without inducing (p)ppGpp (Supplementary Fig. 8). We observed an increase in persistence by about tenfold (Fig. 2b), indicating that GTP depletion is sufficient to generate persisters. Moreover, we performed a non-biased transposon-insertion-sequencing (Tn-seq) analysis in *B. subtilis* to identify genes of which the disruption increased cell survival after prolonged antibiotic treatment (Extended Data Fig. 3). The strongest hits mapped to 12 de novo purine biosynthesis genes in the *pur* operon and *guaA*, encoding GMP synthetase (Extended Data Fig. 3b,c and Supplementary Tables 8 and 9). We verified that disruption of the *pur* operon or *guaA* reduces purine levels, including GTP (Extended Data Fig. 3d), and the corresponding kill curves confirmed increased antibiotic persistence (Fig. 2b,c and Extended Data Fig. 3e). We next wanted to determine whether reducing GTP in the complete absence of (p)ppGpp can still promote persistence. Using a genetic screen, we identified a mutation in *gmk* (encoding guanylate kinase; Methods) that reduces the GTP levels of the (p)ppGpp^0^ strain back to the wild-type level (Extended Data Fig. 4). This mutant (hereafter, (p)ppGpp^0^ *gmk*^*Q110R*^) restored persistence back to wild-type levels (Fig. 2b,c and Extended Data Fig. 4), confirming that GTP depletion is sufficient for antibiotic persistence.

### Single-cell GTP depletion drives persistence uncoupled from tolerance

In addition to increased persistence, we also noted a modest increase in antibiotic tolerance in the GTP-synthesis mutants (Supplementary Fig. 5), consistent with our previous findings that GTP depletion leads to slower growth^35^. Slower growth has been shown to contribute to antibiotic tolerance^11,36^. This raises questions of whether increased antibiotic persistence is simply a consequence of slow growth, and whether it is always correlated with tolerance.

Genetic screens in *B. subtilis* have identified slow-growing mutants, some of which deplete GTP while others do not. We tested a couple of slow-growing mutants that do not rely on GTP depletion. Notably, although these mutants exhibited increased tolerance, they do not show an increase in persistence; in fact, some showed decreased persistence (Fig. 2c). This suggests that, although tolerance and persistence are correlated in certain mutants or conditions^11^, they can be genetically uncoupled.

**Extended Data Fig. 5:**
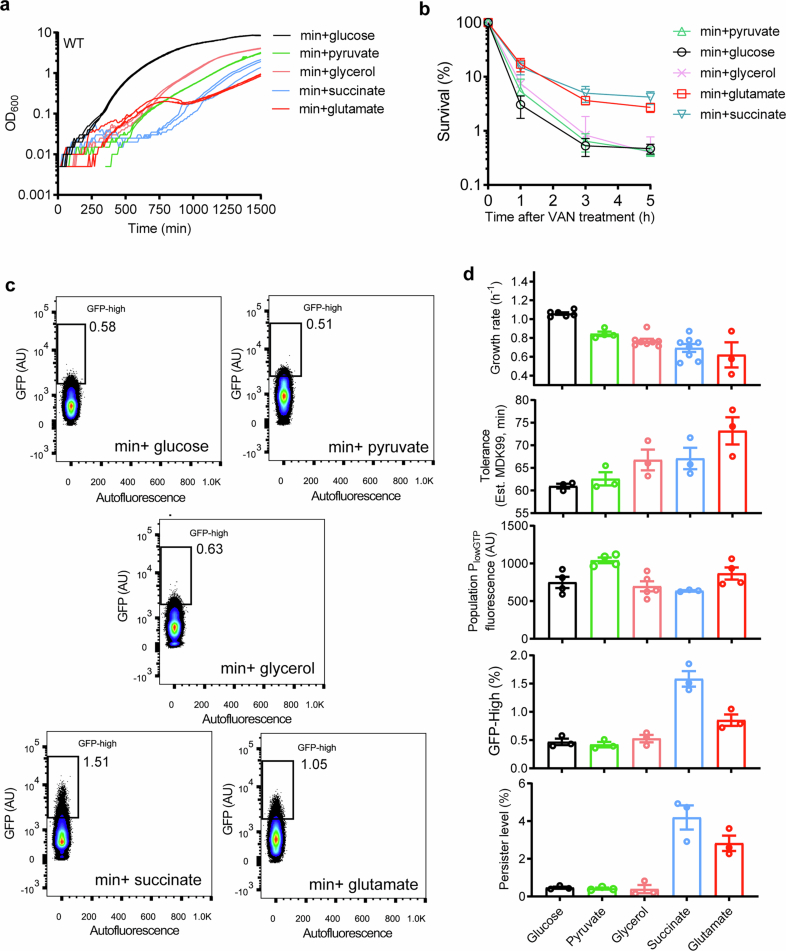
Levels of low GTP cells correlate with persistence in various growth conditions. (**a**) Wild type cells containing the P_lowGTP_ reporter were grown in minimal media with different carbon sources as indicated. Growth was monitored by OD_600_ measurement. Each curve represents a single replicate. (**b**) Survival curves of the populations in (**a**) to vancomycin treatment. (**c**) Distribution of cells with high P_lowGTP_ fluorescence analysed using flow cytometry ( ~ 1.5×10^6^ cells per sample per replicate, three biological replicates). Numbers indicate the fraction (%) of GFP-high cells. The fluorescence cut-off for GFP high cells was selected based on FACS and antibiotic treatment experiment that these high fluorescence cells display persistence phenotype (see Fig. 3a-b). (**d**) Panel of growth rates, tolerance, population P_lowGTP_ fluorescence, frequency of high P_lowGTP_-GFP-high cells, and persistence to vancomycin treatment from the data in **a**-**c**. Values represent mean ± s.d., three biological replicates.

To further distinguish between tolerance and persistence, we grew wild-type cells in different carbon sources, measuring their growth rates and antibiotic killing dynamics (Extended Data Fig. 5a,b). Our results revealed a strong correlation between slower growth rates and increased tolerance (Fig. 2e), but no significant correlation between growth rates and persistence (Fig. 2h). This demonstrates that, although tolerance and persistence can share overlapping characteristics, they represent distinct biological phenomena that can be independently regulated.

Our findings, while unexpected, are explainable: although both slow growth and antibiotic tolerance are population-level phenomena, persistence reflects the behaviour of a phenotypically heterogeneous subpopulation of cells functioning as outliers. To differentiate population-based tolerance from the phenotypically heterogeneous subpopulation of persisters, it is critical to measure GTP levels in single cells.

**Extended Data Fig. 6:**
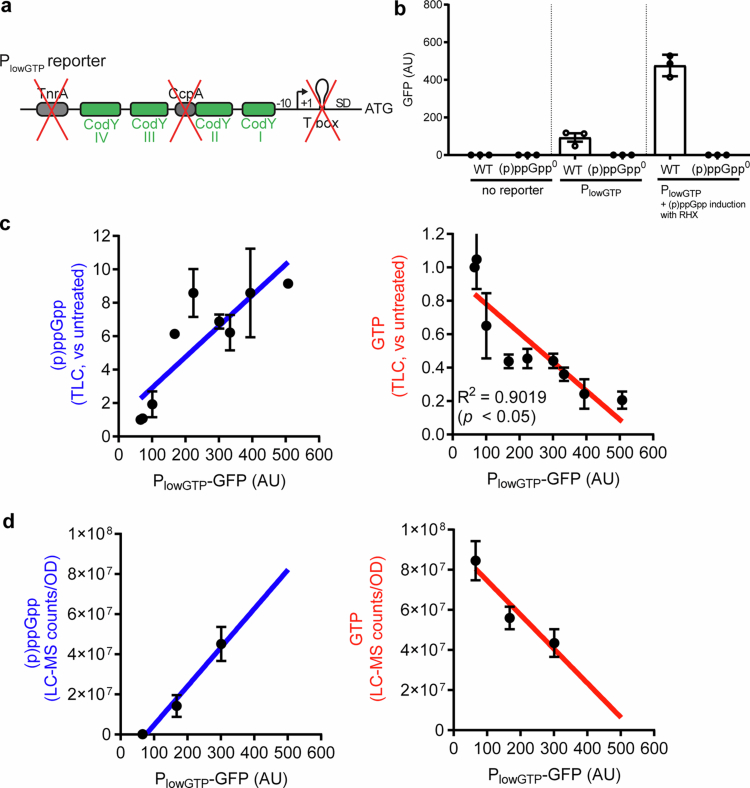
P_lowGTP_ reporter responds to changes in GTP levels. (**a**) Architecture of the P_lowGTP_ reporter derived from the CodY regulatory regions in the *ilvB* promoter. Low GTP inactivates CodY binding thus de-repressing the promoter (that is reporter ON) and vice versa. It has been demonstrated that *ilvB* promoter activity is proportional to GTP over a physiologically relevant range *in vitro*^38^. Our previous transcriptome work showed that the *ilvB* promoter is responding almost exclusively to GTP levels in a variety of conditions and mutants *in vivo*^37^. Sequence of P_lowGTP_ is included in Supplemental Table 4. (**b**) P_lowGTP_ fluorescence in wild type or (p)ppGpp^0^ population during exponential growth in rich media with or without (p)ppGpp induction using arginine hydroxamate (RHX). Background fluorescence was subtracted using strains without the reporter. (**c**-**d**) Correlation of P_lowGTP_ fluorescence versus (p)ppGpp (blue) and GTP (red) in wild type upon (p)ppGpp induction by amino acids starvation. P_lowGTP_ fluorescence was determined using microscopy from averages of > 200 cells each from three biological replicates. AU stands for arbitrary units. (**c**) Nucleotide levels were measured by thin layer chromatography (TLC) and normalized to their levels before induction. R square was derived from linear regression from three biological replicates for each data point. (**d**) Nucleotide levels were measured by LC-MS and normalized to OD_600_. Values represent mean and error bars indicate s.d. from three biological replicates for each data point.

To achieve this, we constructed a fluorescent reporter for GTP levels in single cells. Building on our previous result that the *ilvB* gene is induced by approximately 20-fold after GTP depletion through the CodY repressor—which uses GTP as a co-repressor^37,38^—we cloned the CodY operator from *ilvB* to control fluorescent protein expression ectopically (Extended Data Fig. 6a). This reporter, termed P_lowGTP_ (Fig. 2d), is fully repressed in (p)ppGpp^0^ cells, with fluorescence indistinguishable from background autofluorescence (Extended Data Fig. 6b). In (p)ppGpp^+^ cells, the reporter displays a dose-dependent increase in fluorescence across a physiological range of (p)ppGpp induction and GTP depletion (Extended Data Fig. 6c-d). This response to amino acid or ATP starvation is abolished in (p)ppGpp^0^ mutants (Supplementary Fig. 9). Moreover, neither the P_lowGTP_ reporter nor the CodY regulator interferes with antibiotic survival (Supplementary Fig. 10). The P_lowGTP_ reporter activity is inversely related to the ribosomal P1 promoter activity (Supplementary Fig. 11), which is activated by GTP^35^. Although increased fluorescence could theoretically result from a failure to dilute the fluorescent protein during slow growth, our data show that P_lowGTP_ fluorescence responds to changes in GTP levels rather than slow growth (Supplementary Fig. 12).

After calibrating the P_lowGTP_ reporter in the bulk population, we examined whether it reflects GTP levels in individual cells. Fluorescence-activated cell sorting (FACS) of cells with high P_lowGTP_ fluorescence, followed by analysis using liquid chromatography coupled with mass spectrometry (LC–MS), confirmed that the bright cells had notably lower GTP levels (Supplementary Fig. 13). This demonstrates that the reporter accurately reflects GTP levels at both the population and single-cell levels. Together, these results confirm that our reporter reliably tracks GTP depletion rather than slow growth, enabling us to distinguish between slow-growing tolerant cells and GTP-depleted persister subpopulations.

Using this reporter, we examined GTP distributions in single cells using flow cytometry (Fig. 2d and Supplementary Fig. 14). Most cells are dark during growth in rich medium, consistent with bulk measurements (Extended Data Fig. 6b). However, we identified a heavy-tailed subpopulation of rare, bright cells that depended on the presence of the reporter (Fig. 2d and Supplementary Fig. 14b), indicating substantial GTP depletion. This subpopulation was absent in (p)ppGpp^0^ cells but reappeared in (p)ppGpp^0^ *gmk*^*Q110R*^ cells with rescued persistence (Supplementary Fig. 14c).

**Extended Data Fig. 7:**
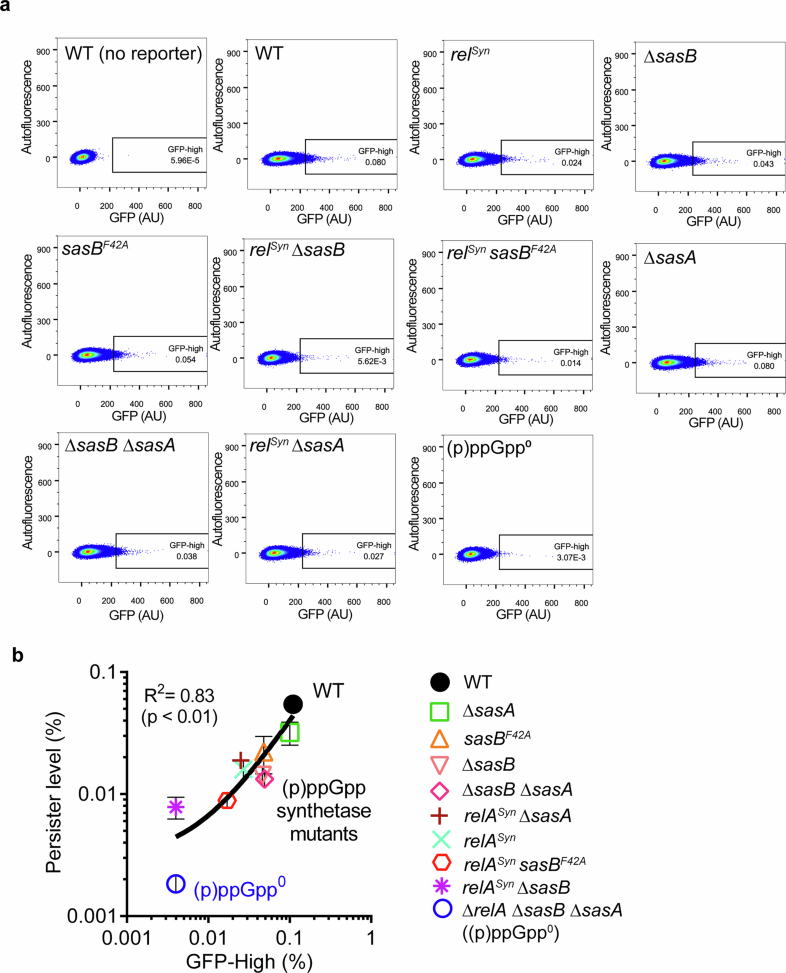
Correlation of low GTP cells and persisters in WT and (p)ppGpp synthetase mutants grew in rich media. (**a**) Flow cytometry analysis of low GTP persisters in exponentially growing populations of wild-type and (p)ppGpp biosynthesis mutants containing the P_lowGTP_ reporter ( ~ 1.5×10^6^ cells each in triplicate). Numbers indicate the fraction (%) of GFP-high cells. ~1.5×10^6^ cells in each sample. The fluorescence cut-off was chosen based on FACS and antibiotic treatment experiment that these high fluorescence cells display persistence phenotype (see Fig. 3a-b). (**b**) Correlation between persisters and GFP-high cells. Persister levels were determined from vancomycin kill curves in Supplemental Data Fig 4. Error bars indicate s.e.m., three biological replicates. Solid line indicates linear regression.

The fractions of bright, low-GTP cells varied when cells were grown in medium with different carbon sources (Extended Data Fig. 5c,d). Notably, the fraction of low-GTP cells was strongly correlated with the fraction of persisters, measured using kill curves (Fig. 2i and Extended Data Fig. 7a,b; 5 h survivors), but was not correlated with the growth rate (Fig. 2g,h,j) or antibiotic tolerance (Fig. 2f and Extended Data Fig. 5d). These results distinguish persistence from tolerance, demonstrating that persistence under various growth conditions is driven by the fraction of low-GTP outliers, not by the population’s average growth rate.

**Fig. 3:**
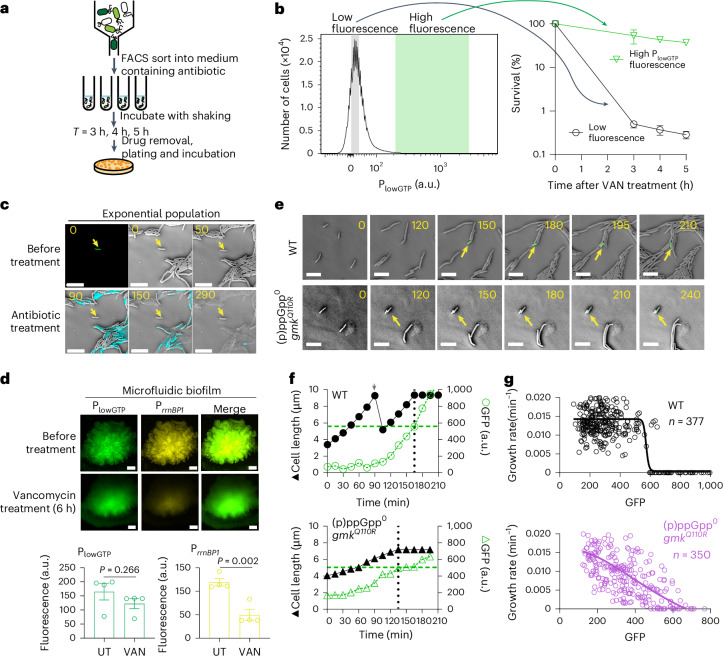
Direct observation of persistence switch in cells through (p)ppGpp–GTP antagonism. **a**, Schematic of the FACS sorting and antibiotic survival assay using the P_lowGTP_ reporter. The bacterial population containing the reporter was sorted based on P_lowGTP_ fluorescence and was immediately treated with antibiotic. Survival was measured by serial dilution and plating followed by colony counting. **b**, WT cells containing the P_lowGTP_–GFP (green fluorescent protein) reporter were FACS-sorted into low or high P_lowGTP_ fluorescence fractions. Approximately 1 × 10^6^ cells were sorted per sample per replicate. The fractions were treated with vancomycin and measured for survival over time. Data are mean ± s.d. from three biological replicates. **c**, Representative time-lapse micrograph from three biological replicates of WT cells containing the P_lowGTP_ reporter in response to antibiotic treatment. Exponentially growing WT cells containing the reporter were patched onto agarose pads made with growth medium. High P_lowGTP_ fluorescence cells were identified (arrow) and monitored for antibiotic survival using carbenicillin and Sytox Blue staining (cyan) for viability. The numbers indicate time in minutes. Scale bar, 10 µm. **d**, The survival of low-GTP cells in biofilm. Representative time-lapse micrograph of WT cells containing both P_lowGTP_ (for low GTP, green) and P_*rrnBP1*_ (for high GTP, yellow) reporters were grown into colony biofilm and then treated with vancomycin for 6 h. Scale bar, 50 µm. The bar graphs indicate changes in the high-GTP and low-GTP populations before and after treatment. Data are mean ± s.d. of four biological replicates. *P* values were calculated using unpaired two-tailed *t*-tests. **e**, Representative single-cell dynamic trace of persister formation. A total of around 30,000 cell growth and division events was recorded to reveal about 10 persistence entrance events. The numbers indicate time in minutes. Scale bar, 10 µm. The arrows indicate a cell with increased P_lowGTP_ fluorescence followed by growth attenuation. **f**, Quantified changes in cell length (left) versus P_lowGTP_ fluorescence (right) from the single trace in **e**. The dotted lines indicate persistence entrance. **g**, Summary data of the specific growth rate and reporter fluorescence from about 100 independent single-cell traces, similar to **e** (ten entrance traces). The solid lines indicate the nonlinear regression model fitted to the data.

Despite the strong correlation between persisters and low-GTP cells, it is essential to determine whether these two subpopulations were indeed the same. To explore this, we used two methods. First, we used FACS to sort exponentially growing wild-type cells containing the P_lowGTP_ reporter (Fig. 3a). FACS-isolated cells with high P_lowGTP_ fluorescence (top 0.1%) were immediately treated with vancomycin. After 5 h of treatment and subsequent antibiotic removal, cells were plated. Around 80% of the sorted low-GTP cells formed colonies compared with only about 0.1% survival in the low-fluorescence population (Fig. 3b). This demonstrates that nearly all low-GTP cells are antibiotic-refractory persisters capable of regrowth after antibiotic removal. We confirmed that this persistence was specifically dependent on P_lowGTP_, using a differentially labelled P_*veg*_ control that measured the effect of protein dilution (Supplementary Fig. 15a), or through fluorescent protein swapping (Supplementary Fig. 15b).

In addition to FACS sorting, we used time-lapse microscopy to monitor cell fate during antibiotic treatment. We found that only bright cells were non-growing before antibiotic treatment and remained viable during the treatment, while the rest of the cells, which elongated and divided, were rapidly killed (Fig. 3c and Supplementary Video 1).

To further investigate persisters in biofilms, where they are commonly found^39^, we developed a microfluidic biofilm system with confocal microscopy (Supplementary Fig. 16). Using dual-labelled cells (P_*rrnBP1*_ and P_lowGTP_), we identified a mixture of persisters and growing cells in microfluidic biofilms (Fig. 3d). After prolonged (6 h) antibiotic treatment, the low-GTP cells survived within the biofilm (Fig. 3d), confirming that the majority of persisters in biofilms are low-GTP cells.

### (p)ppGpp mediates bistable persistence entry through a GTP threshold

**Extended Data Fig. 8:**
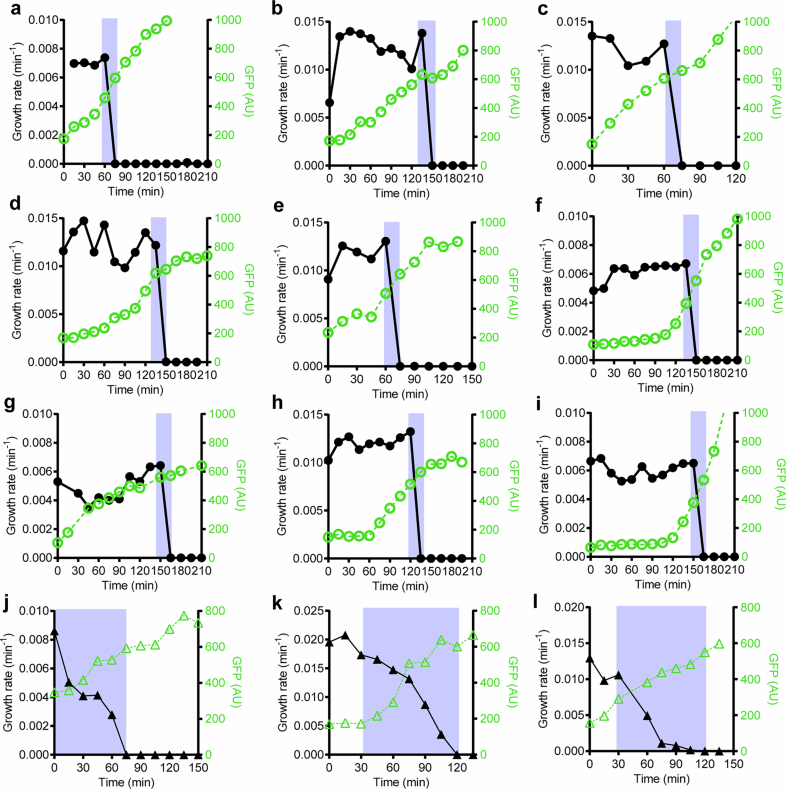
Rapid entrance from growth to persistence in (p)ppGpp^+^ cells. (**a-l**) Single cell traces of spontaneous persistence entrance plotted as growth rate against time in (**a**-**i**) wild type and (**j**-**l**) (p)ppGpp^0^ *gmk*^*Q110R*^ mutant. Changes in P_lowGTP_ reporter fluorescence (green) and growth rate (black) are shown. Persistence entrance in each trace is highlighted by the shaded area. Overall, we observed that, the first event in pre-persister is that P_lowGTP_ reporter fluorescence (reflecting drop in GTP) increases, sometimes over several cell cycle (up to 120 min) while cells are growing at a normal rate without slowing down. Only as the fluorescence reaches a common threshold (on our microscope ~600 fluorescence unit), growth suddenly stopped within our 15 min observation window. If bacteria stop growth first, then we would see fluorescence increase only after growth cessation. This conclusion applies to all our cell trajectory measurements. The results suggest that GTP drop occurs before growth cessation, rather than GTP drops once bacteria stop growing.

After confirming that P_lowGTP_-bright cells are persisters, we investigated how these persisters form before antibiotic treatment. Our goal was to observe how rare, spontaneous persisters emerge from actively growing wild-type cells, and to examine how this process relates to GTP levels. We seeded highly diluted, growing cells onto solid growth medium and monitored around 30,000 growth and division events (Supplementary Fig. 17a,b). From this, we identified ten cells that entered a persister state before reaching a high cell density (Fig. 3e, Supplementary Fig. 17c–k and Supplementary Video 2). We retrospectively analysed the dynamics of their transition into persistence by quantifying their growth, division and GTP reporter activity. This revealed distinct, switch-like behaviours at the single-cell level (Fig. 3f and Supplementary Fig. 17c–k). Initially, each pre-persister cell maintained the same elongation rate as actively growing cells, while its P_lowGTP_ fluorescence gradually increased, often over more than one cell division (Supplementary Fig. 17c–k). This was followed by a sudden stop in elongation, marking entry into dormancy when the P_lowGTP_ fluorescence reached a threshold (Fig. 3f and Extended Data Fig. 8a–i). In all cases, the reporter fluorescence increased before growth inhibition occurred (Fig. 3f and Extended Data Fig. 8a–i), indicating that GTP depletion triggers growth inhibition rather than resulting from it.

The fluorescence thresholds were highly consistent across different persister entry events (Fig. 3g), suggesting that the switch to persistence is activated when intracellular GTP levels fall to a specific threshold. This threshold aligns with values seen in our microscopy and flow cytometry experiments, corresponding to an estimated GTP concentration of around 0.1–0.2 mM (ref. ^40^). This range is comparable to what is observed in wild-type cells experiencing strong (p)ppGpp accumulation (Extended Data Fig. 6c,d), where most cells became starvation-triggered persisters.

While GTP depletion leads to persistence, we next examined the role of (p)ppGpp in persister dynamics. Notably, the (p)ppGpp^0^ *gmk*^*Q110R*^ mutant, which has GTP levels similar to wild-type cells but lacks (p)ppGpp (Fig. 2c), can still form spontaneous persisters (Fig. 3f, Extended Data Fig. 8j–l, Supplementary Fig. 17l–n and Supplementary Video 3). However, these mutants do not exhibit the switch-like dynamics characteristic of persister entry in wild-type cells. Instead, their entry into dormancy is gradual, with elongation slowing as reporter activity increases (Fig. 3f). Moreover, in these mutants, GTP levels and growth rates show a linear relationship (Fig. 3g), contrasting with the switch-like relationship seen in wild-type cells. Thus, (p)ppGpp promotes GTP depletion, enabling the rapid switch-like entry into persistence observed in wild-type cells.

### Lethal and sublethal cell wall antibiotics induce alarmone-mediated persistence

**Fig. 4:**
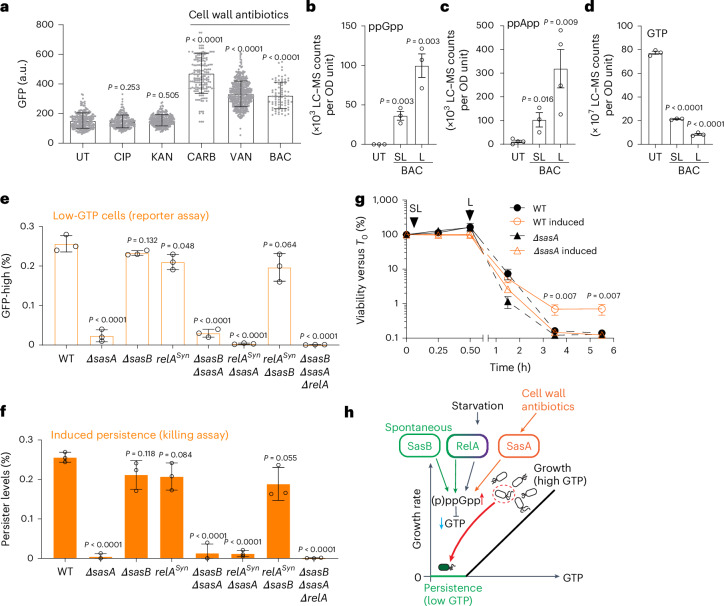
Antibiotic-induced persistence through alarmone accumulation contributes to adaptive survival. **a**, Cell wall antibiotics induce P_lowGTP_ reporter fluorescence. WT cells containing the P_lowGTP_ reporter were treated with 20× MIC ciprofloxacin (CIP), 4× MIC kanamycin (KAN), 200× MIC carbenicillin (CARB), 20× MIC vancomycin (VAN) or 3× MIC bacitracin (BAC) for 2 h. Fluorescence was measured using microscopy (>200 cells each, three biological replicates). **b**–**d**, The levels of ppGpp (**b**), ppApp (**c**) and GTP (**d**) in WT cells treated with sublethal or lethal concentrations of bacitracin for 0.5 h. Sublethal (SL), 0.5× MIC bacitracin; lethal (L), 3× MIC bacitracin. **e**,**f**, The levels of cell wall antibiotic-induced persisters in WT and (p)ppGpp mutants. **e**, Strains containing the P_lowGTP_ reporter before and after 0.5× MIC bacitracin treatment for 0.5 h were measured for generation of low-GTP cells using flow cytometry (around 1 × 10^6^ cells per sample per replicate, three replicates). The levels of induced low-GTP cells were determined by subtracting the levels in uninduced populations. **f**, The survival of WT or (p)ppGpp mutants pretreated with 0.5× MIC for 0.5 h, followed by lethal (3× MIC) bacitracin treatment for up to 5 h. The levels of induced persistence were determined by subtracting the survival of uninduced populations at 5 h. **g**, Survival curve of WT or ∆*sasA* cells pretreated with sublethal (0.5× MIC) bacitracin for 0.5 h, followed by lethal (3× MIC) bacitracin treatment for up to 5 h. For **a**–**g**, data are mean ± s.d. from three or more biological replicates. Statistical comparisons were performed between WT and mutant pairs or untreated and treated pairs using unpaired two-tailed *t*-tests. **h**, Model for persistence pathways mediated by (p)ppGpp-GTP antagonism. Starvation-triggered persistence (purple) is mediated primarily through the (p)ppGpp synthetase Rel. Spontaneous persistence is mediated through the activities of two (p)ppGpp synthetases, Rel and SasB (green). Persisters can also be induced by cell wall antibiotics through SasA (orange). Thus, (p)ppGpp enables integration of different signals to trigger persistence through GTP depletion.

**Extended Data Fig. 9:**
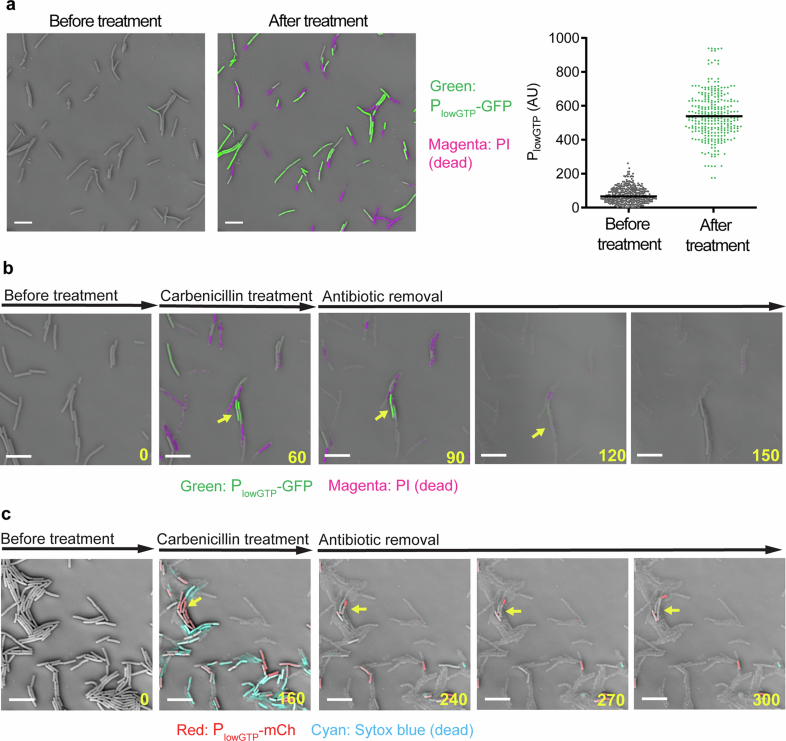
Induction of low GTP persisters by a cell wall antibiotic. (**a**) (Left) Example images of wild type cells containing the P_lowGTP_ reporter before and after treatment with 200x MIC carbenicillin (CARB) and cell death dye propidium iodide (PI, magenta) for 2 h. (Right) Quantitation of P_lowGTP_ fluorescence in survivors (PI-negative cells) before and after treatment. About ~300 survivors were counted. Scale bar: 10 µm. (**b-c**) Representative time-lapse images from three biological replicates of antibiotic-induced survival upon antibiotic treatment. Exponentially growing wild type cells containing the P_lowGTP_-GFP reporter (green) were allowed to grow in the absence of antibiotic followed by carbenicillin treatment with the cell death dye propidium iodide (PI, magenta). Antibiotic was removed by penicillinase treatment. Arrow indicates a cell that regrew after drug removal. Numbers indicate time in min. Scale bar: 10 µm. (**c**) Same experiment as (**b**) but with P_lowGTP_-mCherry reporter (red) and cell death dye sytox blue (cyan). Numbers indicate time in min. Scale bar: 10 µm.

Finally, we used our established system to investigate the poorly understood phenomenon of antibiotic-induced persistence^1^. We found that P_lowGTP_ fluorescence increased in cells exposed to lethal concentrations of cell wall antibiotics, including carbenicillin, vancomycin and bacitracin (Fig. 4a and Extended Data Fig. 9a). While most cells were killed by irreversible damage, a few P_lowGTP_-bright cells survived and resumed growth after the antibiotics were removed (Extended Data Fig. 9b,c and Supplementary Video 4). LC–MS analysis revealed that exposure to both lethal and sublethal doses of bacitracin led to the accumulation of alarmones, such as ppGpp and ppApp, and a decrease in GTP levels (Fig. 4b–d). These results suggest that cell wall antibiotic treatment induces alarmone production and depletes GTP. Using mutants of (p)ppGpp synthetases, we found that a single alarmone synthetase, SasA^21^, is responsible for (p)ppGpp accumulation and GTP depletion during cell wall antibiotic treatment (Fig. 4e and Supplementary Figs. 18 and 19).

To assess whether this alarmone response provides a protective effect, we measured persistence due to treatment with sublethal concentrations (below MIC) of bacitracin (Fig. 4f,g and Supplementary Fig. 20). While sublethal bacitracin did not impact cell viability, it did induce SasA expression^41,42^ (Supplementary Fig. 21). Notably, when followed by a lethal dose of bacitracin (3× MIC), cells pretreated with sublethal antibiotics showed approximately five times more persisters than non-pretreated cells, with minimal differences in tolerance (Fig. 4g). This survival mechanism, dependent on SasA but not on other alarmone synthetases (Fig. 4f), enables pre-exposed cells to better resist lethal antibiotic exposure. Thus, antibiotic-induced persistence may serve as an adaptive strategy, substantially enhancing cell survival against a later lethal antibiotic challenge.

## Discussion

**Extended Data Fig. 10:**
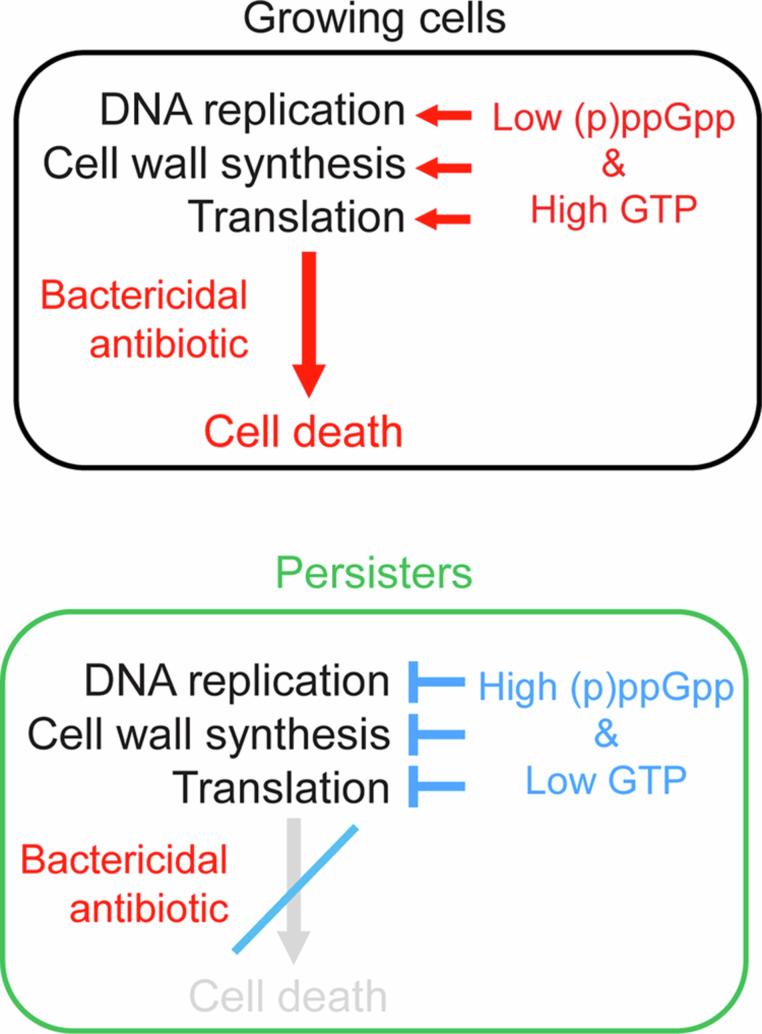
Model for the downstream effects of (p)ppGpp-GTP antagonism leading to persistence. How does low GTP lead to growth suspension and persistence? Although reduction of GTP levels elicits a fundamental change in transcription, the effect of depleting GTP is far beyond transcription. Low GTP is sufficient to broadly suspend diverse growth-determining cellular processes that utilize GTP, including DNA replication, rRNA synthesis, translation initiation and elongation, ribosome assembly, 100S ribosome dissociation, and potentially cell wall synthesis. In addition, since the majority of (p)ppGpp regulation on targets such as protein synthesis, rRNA maturation via ribosome-dependent GTPases, or DNA synthesis all stem from competition between (p)ppGpp and GTP binding to the enzyme active site, low GTP would exacerbate the inhibitory effect of (p)ppGpp on most (p)ppGpp targets. Since lethality of conventional antibiotics stems from deleterious disruption of these growth-associated processes, low GTP bacteria will be able to better survive antibiotic treatment. Therefore, persistence can be due to a global metabolic brake of multiple cellular processes triggered by low GTP. (p)ppGpp-GTP may also facilitate coordinated shut down of macromolecular synthesis, such as transcription and replication, to minimize detrimental conflicts. Finally, the growth suspension may also reduce generation of reactive oxygen species which have been implicated in antibiotic lethality. Contributions of these downstream effects require further investigation.

Our data highlight the pivotal role of (p)ppGpp in persistence. We demonstrate that conditions triggering persistence^10^ also induce (p)ppGpp accumulation, that persistence depends on (p)ppGpp accumulation, and that three (p)ppGpp synthetases are differentially responsible for starvation-triggered, spontaneous and antibiotic-induced persistence (Fig. 4h). While (p)ppGpp senses environmental signals to initiate persistence, GTP antagonism functions as a critical effector, probably through coordinated suspension of multiple GTP-dependent processes such as protein translation and DNA synthesis, as well as potentiating competitive inhibition by (p)ppGpp (Extended Data Fig. 10). Our research also distinguishes persisters from tolerators, revealing that persisters arise through specific genetic pathways that generate individual cells with low GTP, independent of population-wide growth rates or bulk GTP levels.

Our results suggest a metabolic model for spontaneous persistence. Phenotypic switch into persistence can be mediated by competition between toxins and antitoxins in TA modules to provide a threshold-like mechanism^3,12,43^. Our findings suggest that cells can also use an alternative mechanism for the spontaneous entrance, through allosteric enzyme cooperativity and self-amplification of (p)ppGpp synthesis (Supplementary Fig. 22). This enables rapid amplification of a small signal, such as from noise in (p)ppGpp synthesis, to allow spontaneous persister formation in rare individual cells.

Our study also provides strong evidence for bactericidal antibiotic-induced persistence. While previous research suggested that antibiotics induce persistence through cellular damage^15^ and stress responses such as the SOS pathway^44^, our results show that antibiotics, particularly cell-wall-targeting ones, induce persistence through the accumulation of alarmones like (p)ppGpp, driven by SasA expression in response to cell envelope damage^41,42,45^. In the soil bacterium *B. subtilis*, the evolution of this regulation may be shaped by a fitness advantage from its habitat to enhance survival to diffusible antimicrobials from neighbouring microorganisms. For example, bacitracin, which we show to induce persistence, is a diffusible natural antibiotic produced by other soil *Bacillus* species^46^. Clinically, this model may explain why (p)ppGpp mutants in pathogens in the Bacillota phylum, such as *Staphylococcus aureus*, show reduced survival against cell-wall-targeting drugs^47^ and diminished clearance under combination antibiotic therapies^48^.

Finally, persistence may have a key role in the emergence of genetic resistance by expanding the reservoir of surviving bacteria during antibiotic treatment. Given that antibiotic overuse increases bacterial exposure to these drugs, driving the evolution of resistance^49^, targeting persister formation could improve treatment outcomes and help curb the rise of antibiotic-resistant infections.

## Methods

### Bacterial strains and strain construction

A list of all of the bacterial strains, plasmids and oligonucleotides used in this study is provided in Supplementary Tables 1–4. LB and LB agar were used for cloning and maintenance of strains. For selection in *B. subtilis*, media were supplemented with the following antibiotics when required: spectinomycin (80 µg ml^−1^), chloramphenicol (5 µg ml^−1^), kanamycin (10 µg ml^−1^) and tetracycline (10 µg ml^−1^). The combination of lincomycin (12.5 µg ml^−1^) and erythromycin (0.5 µg ml^−1^) was used to select for macrolide–lincosamide–streptogramin (MLS) resistance. Carbenicillin (100 µg ml^−1^) was used for selection in *E. coli*.

*B. subtilis* (p)ppGpp biosynthesis mutants were constructed by transformations of integration plasmids containing an I-sceI endonuclease cut site and regions of homology upstream and downstream of specific synthetase genes (pJW300 for ∆*sasB*; pJW370 for *sasB*^*F42A*^; pJW306 for ∆*sasA*; and pJW371 for *rel*^*D264G*^) followed by transformation of pSS4332 for marker-less recombination^50^. Successful recombination was verified by PCR. For the construction of the (p)ppGpp^0^ mutant, ∆*sasA* ∆*sasB* cells were transformed with ∆*rel*::*mls* PCR product from genomic DNA using the oligos oJW902 and oJW903 followed by MLS resistance selection^26^. Construction of integration plasmid for *sasB*^*F42A*^ was performed using site-directed mutagenesis of pJW370 by PCR using the oligos oJW2309 and oJW2310.

The (p)ppGpp^0^ *gmk*^*Q110R*^ mutant was obtained from isolating suppressor mutants from (p)ppGpp^0^ cells by plating on S7 minimal medium plates containing 1% glucose. The surviving colonies were plated on S7 minimal medium plates containing 0.5% casamino acids and 0.5 mM 8-azaguanine, or S7 minimal medium plates containing 0.5% casamino acids and 0.1 mM guanosine^26^ to differentiate between mutants with mutations in *hprT* or *gmk*. Colonies that can grow on guanosine but not 8-azaguanine were sequenced to identify the mutant *gmk* allele. Whole-genome sequencing was performed to confirm that *gmk*^*Q110R*^ is the only mutation in the strain.

The *guaB*^*down*^ mutant in the (p)ppGpp^+^ background was constructed by transformation of pJW305^26^, which replaces the chromosomal copy of *guaB* with an isopropyl-β-d-1-thiogalactopyranoside (IPTG)-inducible copy of *guaB* (P_*spac*_*-guaB)*. This enables controllable *guaB* expression using IPTG during strain construction and growth to avoid generation of suppressors.

*B. subtilis* deletion mutants were constructed by serial transformation of PCR products from the *B. subtilis* knockout collection (BGSC, Gross laboratory)^51^. Where required, the lox-site-flanked *erm*^R^ or *kan*^R^ cassette was removed using pDR244-*cre* followed by selection for the loss of MLS or Kan resistance.

Construction of P_lowGTP_ fluorescence reporters was done by fusion of PCR products containing the P_lowGTP_ promoter (primers oJW1935 and oJW1936) with coding regions of fluorescence proteins (primers oJW1995 and oJW1996 (for GFP) or oJW2805 and oJW2806 (for mCherry)) using ligase cycling reaction (LCR)^52^. The promoter–fluorescence protein gene fusions were cloned into the pDR110 backbone flanked by *amyE* without the P_*spank*_ promoter for subsequent transformation.

For the construction of the P_*rrnBP1*_-*GFPns* (unstable GFP sequence described previously^53^) reporter, DNA fragments of P_*rrnBP1*_ (primers oJW2083 and oJW2084), *GFPns* (primers oJW1995 and oJW2020), the flanking regions of *lacA* (primers oJW1990 and oJW2414, and oJW2413 and oJW2082) and lox-site-flanked *erm*^R^ cassette (primers oJW2133 and oJW2134) were amplified by PCR using synthetic oligonucleotides or genome DNA. The resulting PCR products were fused by LCR followed by amplification using PCR to generate the linear recombination fragment of *lacA*::P_*rrnBP1*_-*GFPns-lox-ermR-lox* for transformation.

For the construction of P_*sasA*_-*mCh* reporter, DNA fragments of P_*sasA*_ (primers oJW3099 and oJW3079), *mCh* (primers oJW2805 and oJW2806), the flanking regions of *lacA* (primers oJW1990 and oJW2414, and oJW2413 and oJW2082) and lox-site-flanked *erm*^R^ cassette (primers oJW2133/oJW2134) were amplified by PCR using synthetic oligonucleotides or genome DNA. The resulting PCR products were fused by LCR followed by amplification using PCR to generate the linear recombination fragment of *lacA*::P_*sasA*_-*mCh-lox-ermR-lox* for transformation.

For the construction of P_*veg*_*-GFP* reporter, DNA fragments of P_*veg*_ (primers oJW3928 and oJW2806), *GFP* (primers oJW1995 and oJW1996), the flanking regions of *lacA* (primers oJW1990 and oJW2414, and oJW2413 and oJW2082) and lox-site-flanked *erm*^R^ cassette (primers oJW2133 and oJW2134) were amplified by PCR using synthetic oligonucleotides or genome DNA. The resulting PCR products were fused by LCR followed by amplification using PCR to generate the linear recombination fragment of *lacA*:: P_*veg*_*-GFP-lox-ermR-lox* for transformation.

Removal of the lox-site flanked *erm*^*R*^ cassette was done by transformation with pDR244-*cre* and selecting for the loss of MLS resistance. All of the mutants and constructs were verified by DNA sequencing.

### Growth conditions

*B. subtilis* strains were grown in S7 defined medium^54^; MOPS was used at 50 mM rather than 100 mM, supplemented with 0.1% glutamate, 1% glucose and 0.5% casamino acids. Growth of the YB886 strain background was supplemented with 20 µg ml^−1^ tryptophan and 50 µg ml^−1^ methionine. For growth in minimal medium, both glutamate and casamino acids were replaced with 200 µg ml^−1^ l-isoleucine, 200 µg ml^−1^ l-leucine and 200 µg ml^−1^ l-valine, and 1% carbon sources were used as indicated.

Routinely, cells from young colonies on overnight LB-agar plates at 37 °C (<12 h) were inoculated into growth medium and then grown at 37 °C with 250 rpm shaking. Cultures in logarithmic phase (optical density at 600 nm (OD_600_) of around 0.1–0.3) were treated with antibiotics or inducers, including arginine hydroxamate (RHX, 0.5 mg ml^−1^), carbonyl cyanide *m*-chlorophenyl hydrazone (CCCP, 5 µM), sodium azide (NaN_3_, 4 mM) or arsenate (2.5 mM). IPTG was added to a final concentration of 0.5 mM to induce *guaB* expression from an IPTG-inducible promoter (P_*spac*_), while depletion of *guaB* expression was done by omitting IPTG in the growth medium.

The following inducers and concentrations were used unless otherwise specified: RHX, 0.5 mg ml^−1^; CCCP, 5 μM; NaN_3_, 4 mM; arsenate, 2.5 mM; carbenicillin, 0.5 μg ml^−1^ (0.5× MIC) or 100 μg ml^−1^ (200× MIC); bacitracin, 64 μg ml^−1^ (0.5× MIC) or 384 μg ml^−1^ (3× MIC); ciprofloxacin, 0.1 μg ml^−1^ (0.5× MIC) or 4 μg ml^−1^ (20× MIC); kanamycin, 0.625 μg ml^−1^ (0.3× MIC) or 8 μg ml^−1^ (8× MIC); vancomycin, 0.1 μg ml^−1^ (0.5× MIC) or 4 μg ml^−1^ (20× MIC) along with the non-induction controls.

### MIC determination

MICs for chloramphenicol, tetracycline, kanamycin, ciprofloxacin, norfloxacin, rifampicin, bacitracin and vancomycin were determined using the microdilution method^55^. Logarithmic-phase cells were back-diluted to a final titre of around 5 × 10^5^ colony-forming units (CFU) per ml into 96-well plates containing twofold serial dilutions of respective antibiotics in S7_50_ medium with 0.1% glutamate, 1% glucose and 0.5% casamino acids. After incubation for 16–20 h at 37 °C with 250 rpm shaking, the MIC was determined as the lowest drug concentration that prevented visible growth.

### Bacterial growth measurement

For growth measurement, fresh colonies of *B. subtilis* strains on LB agar were resuspended into different growth media as specified and diluted to OD_600_ ≈ 0.005 in 96-well plates. Growth was monitored by OD_600_ at 37 °C under shaking in the Synergy2 microplate reader (BioTek). Doubling times were estimated by fitting the growth data to the exponential growth curve using a custom Python script.

### Persister assay

To prepare exponentially growing *B. subtilis* populations, cells from young colonies on overnight LB-agar plates at 37 °C (<12 h) were inoculated into S7_50_ medium with 0.1% glutamate, 1% glucose and 0.5% casamino acids, and grown to OD_600_ ≈ 0.1–0.3 at 37 °C, 250 rpm. For growth in minimal medium, both glutamate and casamino acids were replaced with 200 µg ml^−1^ l-isoleucine, 200 µg ml^−1^ l-leucine and 200 µg ml^−1^ l-valine, and 1% carbon sources were used as indicated. Treatments with bactericidal antibiotics were performed at the following concentrations: ciprofloxacin, 4 μg ml^−1^ (20× MIC); vancomycin, 4 μg ml^−1^ (20× MIC); kanamycin, 8 μg ml^−1^ (8× MIC); and bacitracin, 384 μg ml^−1^ (3× MIC). To determine cell viability, culture aliquots were taken at *T* = 0 and at designated times after treatment, serially diluted and plated onto LB agar. Plates were incubated at 37 °C overnight. The viability at different timepoints, determined as the CFU per ml and relative survival (versus *T*_0_), was calculated.

For experiments involving pre-induction of cells with (p)ppGpp-inducing agents, cells were grown to OD_600_ ≈ 0.1 and divided into two cultures: one containing the inducing agent (RHX, 0.5 mg ml^−1^; bacitracin, 64 μg ml^−1^; CCCP, 5 μM; NaN_3_, 4 mM; arsenate, 12.5 mM) and other as a non-induction control. The cultures were grown for an additional 30 min under the same conditions (*T* = 0.5 h) and subjected to the persister assay, as described above.

In the case of measuring spontaneous persistence, we defined spontaneous persisters as those that are generated during growth in a non-stressed condition^1,3^. To achieve this, early-exponentially growing cultures were 1:100 back-diluted into fresh medium and regrown to exponential phase (OD_600_ ≈ 0.1–0.3) for one or two rounds followed by antibiotic treatment^1^.

### Estimation of antibiotic tolerance

For the estimation of population tolerance, we used the MDK_99_ (ref. ^1^). To exclude the contribution of persisters to population tolerance, we determined the level of persisters in the biphasic killing curve and subtracted this subpopulation from the bulk population. The MDK_99_ of the population was estimated from the logarithmic killing phase in the killing curve.

### Measurement of intracellular nucleotides using TLC

To measure intracellular nucleotides, cells were first collected from overnight plates, back-diluted to OD_600_ = 0.005 and grown in low-phosphate (0.1× phosphate, 0.5 mM) S7_50_ medium with 0.1% glutamate, 1% glucose and 0.5% casamino acids. Once cultures reached OD_600_ ≈ 0.05, 1 ml cells was labelled with 50 µCi of ^32^P orthophosphate (900 mCi mmol^−1^; Perkin Elmer) for 2–3 generations before treatment or sampling. At OD_600_ ≈ 0.15, RHX, CCCP or arsenate were added to the cultures and the samples were collected at regular timepoints for nucleotide extraction. Nucleotides were extracted by incubating 100 µl cells with 20 µl of 2 M formic acid on ice for at least 20 min. The samples were spotted onto PEI cellulose TLC plates (Millipore, 1055790001) and resolved in 1.5 M or 0.85 M potassium phosphate monobasic (KH_2_PO_4_, pH 3.4) buffer to separate (p)ppGpp or GTP, respectively. TLC plates were exposed on storage phosphor screens (GE Healthcare) and scanned on the Typhoon imager (GE Healthcare).

### Measurement of intracellular nucleotides by LC–MS

LC–MS quantification of nucleotides was performed as described previously^21^. Cells were grown in S7_50_ medium supplemented with 20 amino acids^21^ to OD_600_ ≈ 0.3 at 37 °C, 250 rpm before collection. Then, 25 ml of cultures was sampled and filtered through PTFE membrane (Sartorius, 14555419). For experiments involving bacitracin induction, cells were collected before and after 0.25× MIC (sublethal) or 1.25× MIC (lethal) bacitracin treatment for 30 min. Membranes with cell pellets were submerged in 3 ml extraction solvent mix (on ice 50:50 (v/v), chloroform:water) to quench metabolism, lyse the cells and extract metabolites. Mixtures of cell extracts were centrifuged at 5,000*g* for 10 min to remove organic phase and then centrifuged at 20,000*g* for 10 min to remove cell debris. The samples were analysed using an HPLC–MS system, consisting of a Vanquish UHPLC system linked through electrospray ionization (ESI, negative mode) to the Q Exactive Orbitrap mass spectrometer (Thermo Fisher Scientific) operated in full-scan mode to detect targeted metabolites based on their accurate masses. LC was performed on the Acquity UPLC BEH C18 column (1.7 μm, 2.1 × 100 mm; Waters). The total run time was 30 min with a flow rate of 0.2 ml min^−1^, using solvent A as denoted above and acetonitrile as solvent B. The gradient was as follows: 0 min, 5% solvent B; 2.5 min, 5% solvent B; 19 min, 100% solvent B; 23.5 min, 100% solvent B; 24 min, 5% solvent B; 30 min, 5% solvent B. Quantification of metabolites was performed using MAVEN software^56^ and normalized to the OD_600_ at the time of cell collection.

For LC–MS analysis of FACS-sorted cells, membranes with filtered cells (about 8 × 10^6^ cells) were submerged in 1.5 ml extraction solvent mix (methanol:acetonitrile:H_2_O, 40:40:20) to quench metabolism, lyse the cells and extract metabolites. The cell extract was centrifuged at 21,000*g* for 10 min at 4 °C, and 1 ml of supernatant was then transferred to a new microcentrifuge tube and dried completely with the SpeedVac. Dried metabolites were then resuspended in 100 μl solvent A (97:3 (v/v) water:methanol, 10 mM tributylamine ~pH 8.2–8.5 adjusted with ~9 mM acetic acid). The samples were analysed using an HPLC-MS system consisting of a Vanquish UHPLC system linked through heated electrospray ionization (ESI, negative mode) to a hybrid quadrupole high-resolution mass spectrometer (Q-Exactive Orbitrap, Thermo Fisher Scientific) operated in full-scan selected ion monitoring (MS-SIM) mode to detect targeted metabolites on the basis of their accurate masses. MS parameters were set to a resolution of 140,000, an automatic gain control (AGC) of 3 × 10^6^, a maximum injection time of 100 ms and a scan range of 400–1,000 *m*/*z*. Only ions with a retention time of 10–15 min were scanned by MS. LC was performed on the Acquity UPLC BEH C18 column (1.7 μm, 2.1 × 100 mm; Waters). The total run time was 30 min with a flow rate of 0.2 ml min^−1^, using solvent A and 100% acetonitrile as solvent B. The gradient was as follows: 0 min, 5% B; 2.5 min, 5% B; 19 min, 100% B; 23.5 min, 100% B; 24 min, 5% B; and 30 min, 5% B. Raw output data from the MS was converted to mzXML format using custom software. Quantification of metabolites was performed using MAVEN software^56^ and normalized to an internal standard of six most represented nucleotides detected in the sample.

### Fluorescence microscopy

To monitor (p)ppGpp induction using fluorescence reporters, cells were grown to OD_600_ ≈ 0.1–0.3 followed by 30 min induction with the following inducers at concentrations listed below unless otherwise specified: RHX, 0.5 mg ml^−1^; CCCP, 5 μM; NaN_3_, 4 mM; arsenate, 2.5 mM; carbenicillin, 0.5 μg ml^−1^ (0.5× MIC) or 100 μg ml^−1^ (200× MIC); bacitracin, 64 μg ml^−1^ (0.5× MIC) or 384 μg ml^−1^ (3× MIC); ciprofloxacin, 0.1 μg ml^−1^ (0.5× MIC) or 4 μg ml^−1^ (20× MIC); kanamycin, 0.625 μg ml^−1^ (0.3× MIC) or 8 μg ml^−1^ (4× MIC); vancomycin, 0.1 μg ml^−1^ (0.5× MIC) or 4 μg ml^−1^ (20× MIC), along with the non-induction controls.

All of the imaging samples were spotted onto 1.5% agarose pads made with the same growth medium, and immediately imaged on the Olympus IX-83 inverted microscope (Olympus) using a ×60 phase-contrast objective with fluorescence filters (excitation: 470/20 nm, dichroic mirror: 485 nm, emission: 515/50 nm for GFP; excitation: 575/20 nm, dichroic mirror: 595 nm, emission: 645/90 nm for mCherry or propidium iodide; and excitation: 427/10 nm, dichroic mirror: 595 nm, emission: 472/30 nm for Sytox Blue). Metamorph Advanced (v.7.8.3.0) (Molecular Devices) was used for microscopy data collection. Single-cell time-lapse imaging was performed at 15 min intervals for each field at 37 °C using a temperature-controlled imaging chamber (Tokai Hit) coupled to an automatic stage and the microscope control as described previously^57^. The measurement was generally over the course from the birth of the cell until the time lapse stopped owing to crowding of the microcolony or, in rare cases, severe drifting of focus. When comparing phenotypes between strains, both strains were imaged in parallel on the same imaging dish using the same microscope with same settings. For imaging persister survival in time-lapse experiments, final concentrations of 5 μg ml^−1^ carbenicillin (10× MIC) and 200 nM Sytox Blue or propidium iodide (Molecular Probes) were applied to the agarose pads at designated times. To remove carbenicillin after treatment, 5 U ml^−1^ final concentration of penicillinase (Sigma-Aldrich, P0389) was applied. Strains without the fluorescence reporters were used for autofluorescence measurement.

### Biofilm growth and imaging

*B. subtilis* biofilms were grown on a custom microfluidic device fabricated with polydimethylsiloxane. The device contains a central chamber connected to inlet and outlet media channels, allowing for constant medium flow through the central chamber. A semipermeable dialysis membrane was fixed on top of the central chamber to provide a platform for biofilm growth. This setup allows diffusion of nutrients or small molecules from the medium flow underneath to support biofilm growth on the membrane and allows subsequent treatment with antibiotics. To grow *B. subtilis* biofilms, 1 µl of early exponential phase culture (OD_600_ ≈ 0.05) was applied onto the membrane and grown at room temperature (25 °C) for 24 h under a constant flow of S7_50_ medium supplemented with 0.5% glutamate and 0.5% glycerol.

Imaging of biofilms was performed with the IXplore SpinSR confocal imaging microscope (Olympus) using a ×20 phase-contrast objective with fluorescence filters (488 nm laser with 510–550 nm emission filter for GFP; 561 nm laser with 575–625 nm emission filter for mCherry). Biofilms at 24 h after inoculation were imaged before and after switching to the same growth medium containing 4 µg ml^−1^ vancomycin. In total, 51 stacks at 1 µm intervals were taken for each timepoint. Images were projected along the *z* axis from the top of the biofilm using maximum-intensity projection using cellSense Dimension v.2.2 (Olympus). For quantitation of reporter signals within the biofilm, we sampled around four random regions within the centre of the biofilm and measured their GFP and mCherry intensities. The background fluorescence was measured from regions without biofilm and used for background subtraction.

### Flow cytometry and cell sorting

Flow cytometry was performed at the UWCCC flow cytometry core. To prepare samples for flow cytometry, cells from young colonies on overnight LB-agar plates at 37 °C (<12 h) were inoculated into and grown in S7_50_ medium with 0.1% glutamate, 1% glucose and 0.5% casamino acids to OD_600_ ≈ 0.1–0.3 at 37 °C, 250 rpm. For growth in minimal medium, both glutamate and casamino acids were omitted and 1% carbon sources were used as indicated. Cells were immediately fixed with 0.4% paraformaldehyde for 15 min at room temperature, washed three times with 1× PBS and kept at 4 °C until analysis. Fixation was verified by viability plating and microscopy. Flow cytometry analysis was performed using the BD LSRFortessa flow cytometer (BD Biosciences) with a 70 µm nozzle. BD FACSDiva v.8.0.2 was used for data collection. Cell populations were detected using both forward and side scatter (FSC and SSC). Single-cell fluorescence was measured using the 488 nm laser and detection filters for GFP (530/30 nm, 505LP dichroic filter). Autofluorescence was measured by analysing parental strains without the fluorescence reporter and subtracted from the raw reporter fluorescence. Approximately 1.5 million events were measured for each sample. For the determination of antibiotic-induced persistence, the frequency of low-GTP cells after antibiotic induction was subtracted from the frequencies before induction.

FACS was performed at the UWCCC flow cytometry core. To prepare samples for cell sorting, cells were collected from young colonies on overnight LB-agar plates at 37 °C (<12 h) and grown in S7_50_ medium with 0.1% glutamate, 1% glucose and 0.5% casamino acids to OD_600_ ≈ 0.3 at 37 °C, 250 rpm. FACS analysis was performed using the BD FACSAria cell sorter (BD Biosciences) with a 70 µm nozzle at room temperature using the 488 nm laser and 530/30 nm detection filters for GFP, and the 561 nm laser and 610/20 nm detection filters for mCherry. BD FACSDiva v.8.0.2 was used for data collection. Autofluorescent cells were eliminated by gating using an isogenic strain without the fluorescent reporters. At least 1,000 cells were obtained from the rarest gate for each sample. Cell recovery rate was estimated to be >90% based on viability counting on LB plates. For antibiotic treatment, cells were directly sorted into tubes containing 4× MIC of vancomycin followed by shaking at 37 °C. Aliquots were taken at different times for serial dilution and plating to measure survival by colony counting. The number of cells before treatment (*T*_0_) was measured using the cell sorter.

For FACS-sorting of cells for LC–MS analysis, cells containing the P_lowGTP_ reporter were grown in S7_50_ medium with 0.1% glutamate, 1% glucose and 0.5% casamino acids to either exponential (OD_600_ ≈ 0.2) or stationary (OD_600_ ≈ 4.0) phase. Both populations were mixed at a 10:1 ratio, and immediately FACS-sorted into low-fluorescence or high-fluorescence fractions. Autofluorescent cells were eliminated by gating using an isogenic strain without the fluorescent reporters. Approximately 8 × 10^6^ cells were sorted and filtered on the PTFE membrane (Sartorius, 14555419) to remove the sheath fluid. Membranes with filtered cells were then subjected to metabolite extraction and LC–MS analysis. CFU count analysis was performed from a small aliquot of the sorted fractions to account for potential variations in the number of cells sorted between fractions.

### Tn-seq

The *B. subtilis* 168 transposon mutant library was provided by the Grossman laboratory^58^. Construction of the library was performed as follows. In brief, in vitro transposition of *B. subtilis* 168 genomic DNA (gDNA) with magellen6x transposon was performed by mixing 1.3 µg pCJ41 (containing magellen6x transposon), 34 ng purified MarC9 transposase, 5 µg *B. subtilis* gDNA, 10 µl 2× buffer A (41 mM HEPES pH 7.9, 19% glycerol, 187 mM NaCl, 19 mM MgCl_2_, 476 µg ml^−1^ BSA and 3.8 mM DTT) into a 20 µl reaction in vitro and incubated overnight at 30 °C. The transposed DNA was precipitated and resuspended in 2 µl 10× buffer B (500 mM Tris-Cl pH 7.8, 100 mM MgCl_2_, 10 mM DTT), 2 µl 1 mg ml^−1^ BSA and 11 µl H_2_O followed by 4 h incubation at 37 °C. After incubation, 4 µl of 2.5 mM dNTPs and 1 µl of 3U µl^−1^ T4 DNA polymerase were added to the DNA and further incubated for 20 min at 12 °C, followed by heat-inactivation at 75 °C for 15 min. Next, 0.2 µl 2.6 mM NAD and 1 µl of 10 U µl^−1^ *E. coli* DNA ligase were added and the reaction was incubated overnight at 16 °C. The resulting in vitro transposed and repaired gDNA was transformed into *B. subtilis* 168 and plated onto LB agar containing spectinomycin and incubated overnight. Colonies containing the transposon were washed off and pooled into a single library. The library was estimated to contain around 50,000 unique transposon inserts across the genome.

For the selection experiment with the transposon library, an aliquot of the library was inoculated and grown in S7_50_ medium with 0.1% glutamate, 1% glucose and 0.5% casamino acids supplemented with tryptophan (20 µg ml^−1^) at 37 °C, 250 rpm. At OD_600_ ≈ 0.1–0.3, the cultures were treated with 20× MIC vancomycin or ciprofloxacin. Cultures before and after antibiotic treatment were plated onto LB plates and recovered after incubation for around 14 h at 37 °C. Around 650,000 colonies from each sample were pooled and snap-frozen for gDNA extraction and sequencing library preparation.

Preparation of the sequencing library was performed as previously described^59^. Frozen cell pellets were resuspended in 500 µl lysis buffer with lysozyme and RNase A (20 mM Tris-HCl pH 7.5, 50 mM EDTA, 100 mM NaCl, 2 mg ml^−1^ lysozyme, 120 µg ml^−1^ RNase A) and incubated at 37 °C for 20–30 min. Next, the incubated cell lysate was mixed with 60 µl 10% *N-*lauroylsarkosine and further incubated at 37 °C for 15 min. gDNA was purified using 600 μl phenol, then 600 µl phenol:chloroform:isoamyl alcohol (25:24:1) and finally 600 µl pure chloroform. DNA in the aqueous phase was precipitated using 1/10 volumes of 3 M NaOAc and 2 volumes of 100% ethanol. The DNA pellet was washed with 70% ethanol, air-dried on the bench and resuspended in 10 mM Tris-HCl, pH 8.5 and stored at 4 °C. For each sample, 6 µg of DNA was used for MmeI digestion in 200 µl (6 µg gDNA, 6 µl MmeI (2,000 U ml^−1^, NEB), 0.5 µl 32 mM *S*-adenosylmethionine, 20 µl NEB CutSmart Buffer and double-distilled H_2_O up to 200 µl). DNA was digested for 2.5 h at 37 °C, after which 2 µl calf intestinal phosphatase (10,000 U ml^−1^, NEB) was added to the digest and the sample was incubated for 1 h at 37 °C. Digested gDNA was extracted with 200 µl phenol:chloroform:isoamyl alcohol (25:24:1) followed by 200 µl of pure chloroform. DNA in the aqueous phase was first mixed with 1/10 volume of 3 M NaOAc and 67 ng ml^−1^ glycogen, and then 2.5 volumes of 100% ethanol. The tubes were then placed at −80 °C for 20 min and then centrifuged at maximum speed for 15 min at 4 °C. Precipitated DNA was washed with 150 µl 70% ethanol twice at room temperature, air-dried and resuspended in 15 µl of double-distilled H_2_O.

For annealing of the DNA adaptor, 20 µl of 100 µM synthesized oligos (IDT) were mixed with 1 µl of 41 mM Tris-HCl pH 8.0 (final concentration of adaptor, 50 µM in 1 mM Tris-HCl pH 8.0). Oligos were annealed by heat denaturation (95 °C for 5 min) and stepwise cool-down (94 °C for 45 s then repeat with −0.3 °C per cycle for 250 cycles, then hold at 15 °C) using a PCR machine. Annealed adaptors were diluted to 3.3 µM in double-distilled H_2_O and stored at −20 °C.

For adaptor ligation, 5 µl of digested DNA was mixed with 1 µl of 3.3 µM DNA adaptor, 1 µl of 10× T4 DNA ligase buffer (NEB), 1 µl of T4 DNA ligase (400,000 U ml^−1^, NEB) and 2 µl double-distilled H_2_O. The ligation mix was incubated overnight at 16 °C in a PCR machine.

Amplification of the adaptor-ligated DNA library was performed using barcoded primers and Phusion high-fidelity DNA polymerase (NEB) for 18 cycles according to the provided instructions. The PCR products were mixed in equal amounts, purified by size exclusion and submitted for sequencing using the Illumina sequencing primer (5′-ACACTCTTTCCCTACACGACGCTCTTCCGATCT-3′). Deep sequencing was performed on the Illumina HiSeq 2500 (Illumina) system by the University of Michigan DNA Sequencing Core. Analysis of sequencing data was performed using a custom Python script and mapped to the *B. subtilis* 168 reference genome (NCBI: NC_000964.3). Visual inspection of transposon insertion profiles was done using GenomeBrowse (Golden Helix).

### Expression and purification of *B. subtilis* Rel

The plasmid for Rel purification was constructed as follows. The *B. subtilis rel* coding sequence was PCR amplified from NCIB3610 genomic DNA using the primers oJW3196 and oJW3197. The pE-SUMO expression vector was amplified using the primers oJW3194 and oJW3195. The PCR products were assembled to generate pJW753 by Golden Gate assembly (New England BioLabs). Plasmids were verified by DNA sequencing.

To express His_6_–SUMO–Rel, fresh transformants of *E. coli* BL21 carrying pE-SUMO-*rel* were grown in LB at 37 °C to OD_600_ ≈ 0.5, followed by 1:50 dilution into Terrific Broth and grown at 30 °C until OD_600_ ≈ 1.5. His_6_–SUMO–Rel expression was induced with 1 mM IPTG for 4 h at 30 °C. Cells were pelleted and stored at −80 °C until use. Frozen cell pellets were thawed on ice, resuspended in ice-cold lysis buffer (50 mM Tris-HCl, pH 8, 1 M NaCl, 10 mM imidazole, DNase and cOmplete protease inhibitor (Roche)), and lysed using a French press at 4 °C. The cell lysate was centrifuged at 4 °C, 16,000*g* for 30 min to obtain the supernatant. The filtered supernatant was injected into the ӒKTA FPLC system (GE Healthcare) and passed through the HisTrap FF column (GE Healthcare). His_6_–SUMO–Rel was eluted with a gradient of buffer A (50 mM Tris-HCl, pH 8, 1 M NaCl, 5% glycerol, 10 mM imidazole) and buffer C (50 mM Tris-HCl, pH 8, 1 M NaCl, 5% glycerol, 500 mM imidazole). The fractions containing the protein were pooled with 300 µl SUMO protease into Spectra/Por dialysis tubing (Spectrum), and dialysed into 50 mM Tris-HCl, 1 M NaCl, 1 mM β-mercaptoethanol and 5% glycerol overnight. Rel without the His_6_–SUMO tag was passed through the HisTrap FF column and then purified by size exclusion using the Superose 12 10/300 GL column (GE Healthcare) with the ӒKTA FPLC system. The fractions containing the Rel protein were pooled and measured for its concentration using the Bradford Assay (Bio-Rad). Aliquots were snap-frozen using liquid nitrogen and stored at −80 °C.

### In vitro pppGpp synthesis assay

In vitro pppGpp synthesis by Rel was monitored by measuring synthesis of radiolabelled pppGpp over time. The reaction contained 236 nM *B. subtilis* Rel, 0.05 µM [α^32^P]GTP, 1 mM ATP, 50 mM NaCl and 10 mM ATP in 20 mM Tris-HCl pH 7.5, with or without 10 µM non-radioactive pppGpp. The reaction lacked manganese to avoid a potential effect of pppGpp hydrolysis. The reaction was initiated by the addition of ATP and incubated at 37 °C. At the indicated times, 10 µl of the reaction was mixed with 2 µl of 2 M formic acid and chilled on ice for 20 min to quench the reaction. Then, 1 µl samples were spotted onto PEI cellulose TLC plates (Millipore, 1055790001) and resolved in 1.5 M potassium phosphate monobasic (KH_2_PO_4_, pH 3.4) buffer to separate pppGpp. TLC plates were dried and exposed on storage phosphor screens (GE Healthcare) and scanned on the Typhoon imager (GE Healthcare).

### Quantification and statistical analysis

For TLC experiments, nucleotide spots were quantified using ImageQuant v.5.0 (Molecular Dynamics). The raw intensities were corrected to the number of phosphates in the corresponding nucleotide and normalized to OD_600_ or ATP level before treatment (ATP_*T*=0_) for comparison between samples. For in vitro pppGpp synthesis assays, changes in pppGpp levels were normalized to *T* = 0.

Microscopy image analysis and cell parameters (cell area and fluorescence intensity) measurements were performed using Metamorph Advanced (v.7.8.3.0) (Molecular Devices). Background and autofluorescence were subtracted by comparing images obtained from identical strains without the fluorescence reporter. The single-cell specific growth rate (*µ*) at each frame was calculated using the equation $\mu =\frac{1}{l}\frac{\Delta l}{\Delta t}$, where *l* is the cell length, Δ*l* is the change in cell length and Δ*t* is the change in time (in min). Flow-cytometry data were analysed using FlowJo X (FlowJo); an example gating strategy is shown in Supplementary Fig. 14a. Cells within a narrow range of cell sizes were gated, subgated to filter cell aggregates and then measured for their fluorescence distribution. Autofluorescence was measured using isogenic strains without the fluorescent reporters and subtracted from raw reporter signals. Gating for P_lowGTP_-high cells was set at fivefold or higher above mean population fluorescence. This cut-off agrees with our FACS sorting experiment in which cells with reporter fluorescence above this threshold are predominantly persisters. For the determination of antibiotic-induced persistence, the frequency of low-GTP cells after antibiotic induction was subtracted from the frequencies before induction.

Statistical information of individual experiments is included in the figure legends. *n* represents the number of biological replicates or number of cells for experiments involving single-cell measurements as indicated in the legends. Significance was tested using unpaired two-tailed *t*-test or as specified in the figure legend. Confidence intervals were calculated based on binomial distribution. Prism 7 (GraphPad) was used for statistical analysis.

### Reporting summary

Further information on research design is available in the Nature Portfolio Reporting Summary linked to this article.

## Supplementary information

Supplementary Information Supplementary Figs. 1–22 and Supplementary Tables 1–9.

Reporting Summary

Supplementary Video 1 Antibiotic survival of low-GTP cells.

Supplementary Video 2 Spontaneous persistence entrance in wild-type *B. subtilis.*

Supplementary Video 3 Spontaneous persistence entrance in (p)ppGpp^0^ *gmk*^*Q110R*^ mutant.

Supplementary Video 4 Antibiotic-induced persistence in single cells.

## Data Availability

Data generated in this study are included in the figures and Supplementary Information as much as possible. The sequencing data used for Tn-seq analysis are available at SRA under BioProject under accession number PRJNA1229644. Any other relevant data supporting the findings of this study are available from the corresponding author on request.
