# Supplementary material for: A shared alarmone–GTP switch controls persister formation in bacteria
Source: Nat Microbiol. 2025 May 15;10(7):1617–29. doi: 10.1038/s41564-025-02015-6 (PMC12221987; doi:10.1038/s41564-025-02015-6)
Supplement: Supplementary file 1 — Supplementary Figs. 1–22 and Supplementary Tables 1–9. [file 41564_2025_2015_MOESM1_ESM.pdf]

nature microbiology

Supplementary information

https://doi.org/10.1038/s41564-025-02015-6

A shared alarmone–GTP switch controls persister formation in bacteria

In the format provided by the authors and unedited

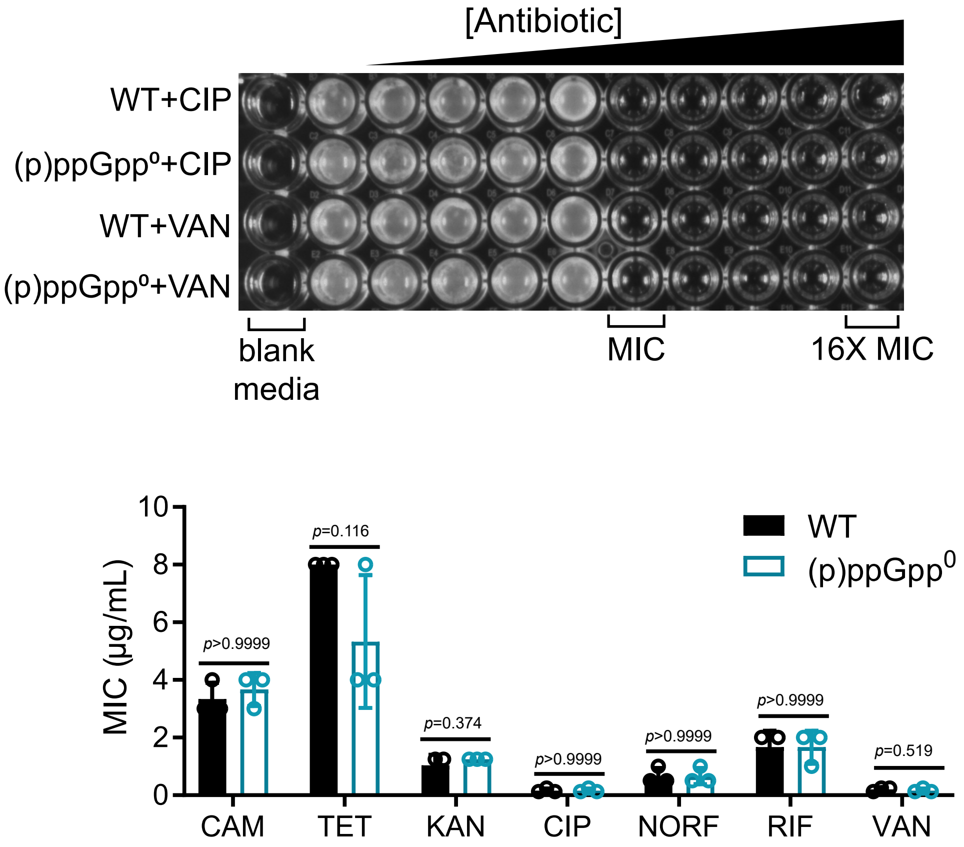

	CAM	TET	KAN	CIP	NORF	RIF	VAN
WT	3	8	1.0±0.2	0.2±0.04	0.7±0.2	1.7±0.3	0.2±0.04
(p)ppGpp^0	3	5.3±2.31	1.25	0.2±0.04	0.7±0.2	1.7±0.3	0.2±0.04

Supplemental Data Fig. 1. WT and (p)ppGpp-null mutant have similar drug resistance as defined by minimal inhibitory concentrations (MICs). a) Minimal inhibitory concentrations (MICs) of wild type (WT) and (p)ppGpp^0 mutant to chloramphenicol (CAM), tetracycline (TET), kanamycin (KAN), ciprofloxacin (CIP), norfloxacin (NORF), rifampicin (RIF) and vancomycin (VAN). Values represent mean and errors represent s.d., three biological replicates. *p* values were derived from unpaired two-tailed t-test.

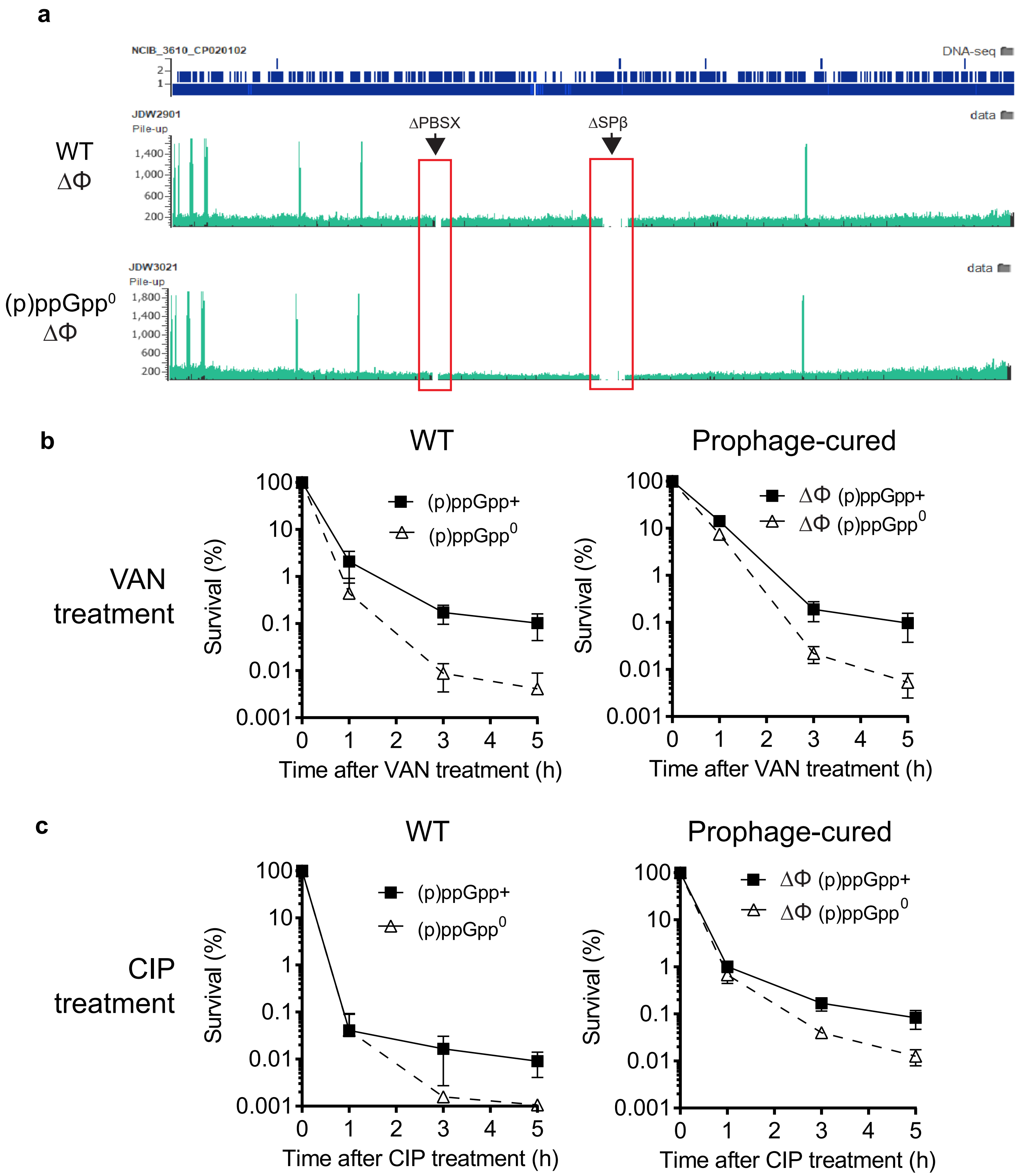

Supplemental Data Fig. 2. Effect of (p)ppGpp on persistence is independent of prophages. (a) Whole genome sequencing of the prophage-cured wild type and (p)ppGpp^0 strains confirming the loss of prophages. (b-c) Survival curves of (p)ppGpp+ and (p)ppGpp^0 strains in either wild type or prophage-cured background (ΔΦ for Δ*zpdN* ΔSPβ ΔPBSX) after treatment with (b) 20x MIC vancomycin (VAN) or (c) ciprofloxacin (CIP). Values represent mean and error bars represent s.d., three biological replicates.

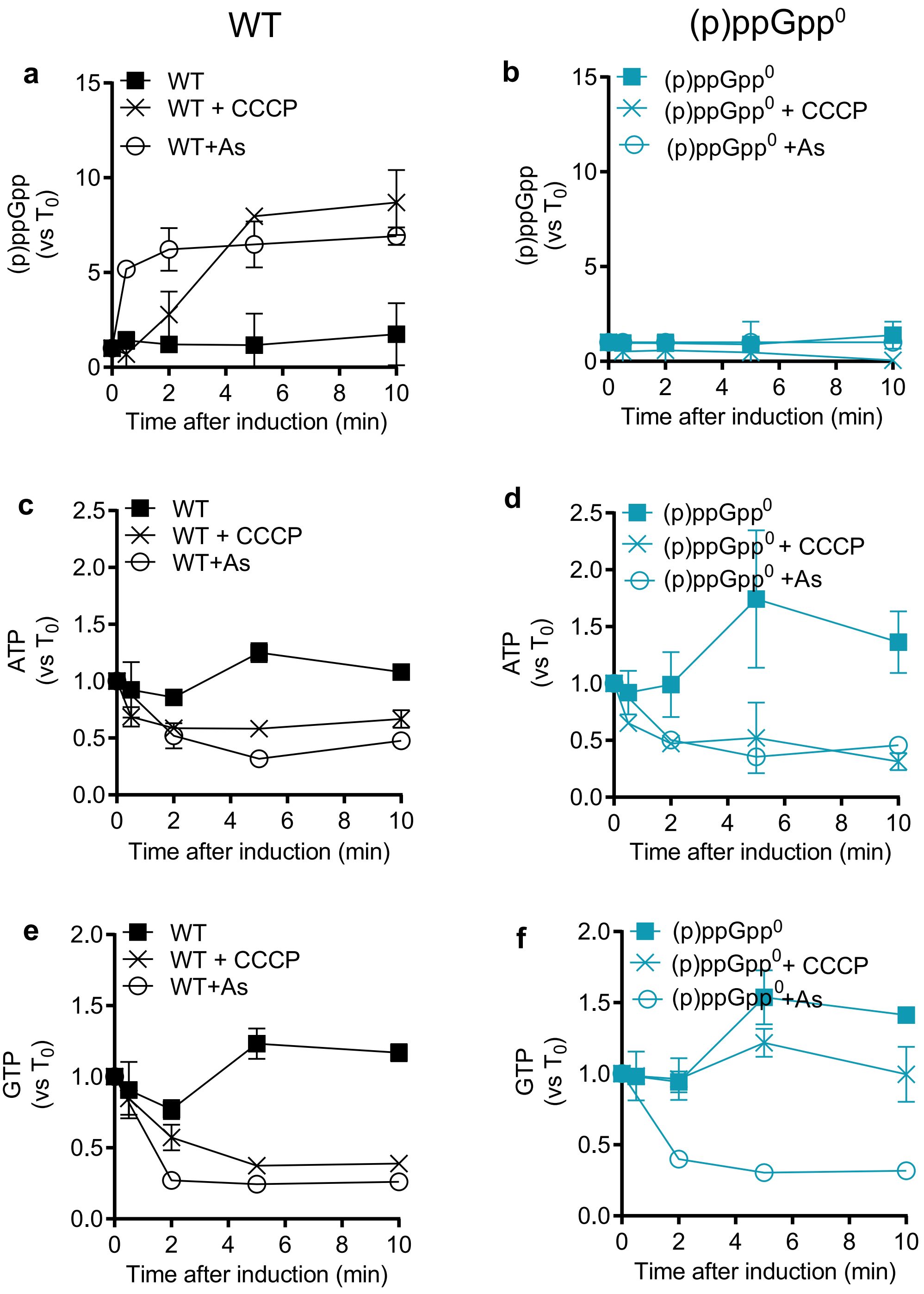

Supplemental Data Fig. 3. Treatment with ATP synthesis inhibitors induces (p)ppGpp and reduces GTP. (a-b) Changes in (p)ppGpp levels in WT and (p)ppGpp⁰ cells treated with carbonyl cyanide 3-chlorophenylhydrazone (CCCP) or arsenate (As). (c-d) Changes in ATP levels in WT and (p)ppGpp⁰ cells treated with CCCP or As. (e-f) Changes in GTP levels in WT and (p)ppGpp⁰ cells treated with CCCP or As. Nucleotide levels were measured by thin layer chromatography (TLC) and normalized to their levels before induction (T_0). Values represent mean and error bars represent s.d., three biological replicates.

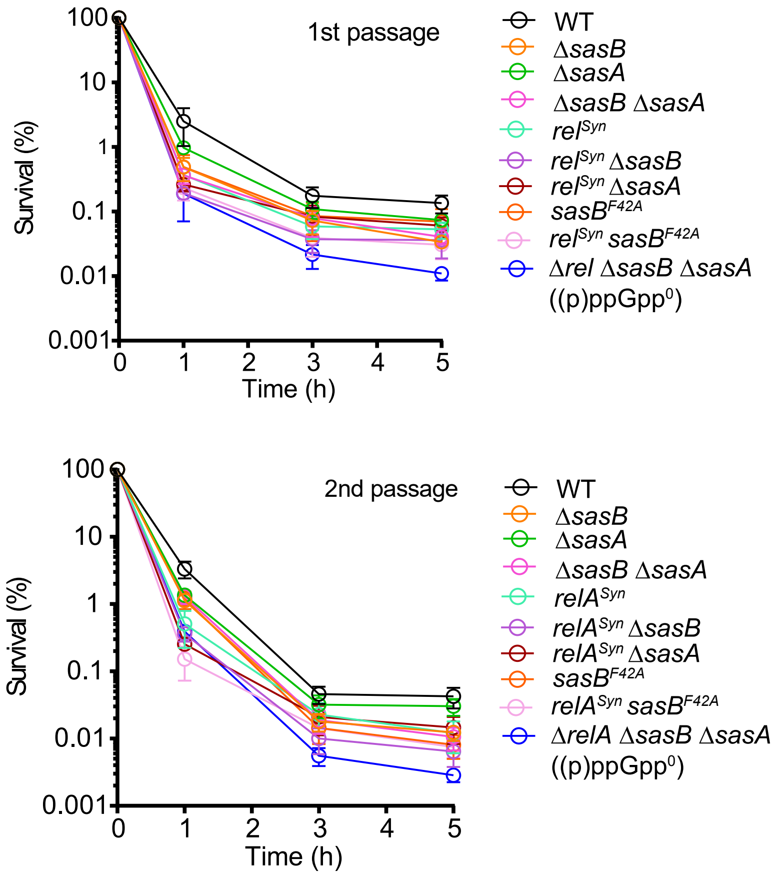

Supplemental Data Fig. 4. Spontaneous persistence in wild type and different (p)ppGpp synthetase mutants. Vancomycin kill curves of exponential phase passaged wild type (WT) and (p)ppGpp synthetase mutants (1st and 2nd passages). Values represent mean ± s.e.m., three biological replicates. See also Supplemental Data Table 6 for a summary table of persister levels and statistics.

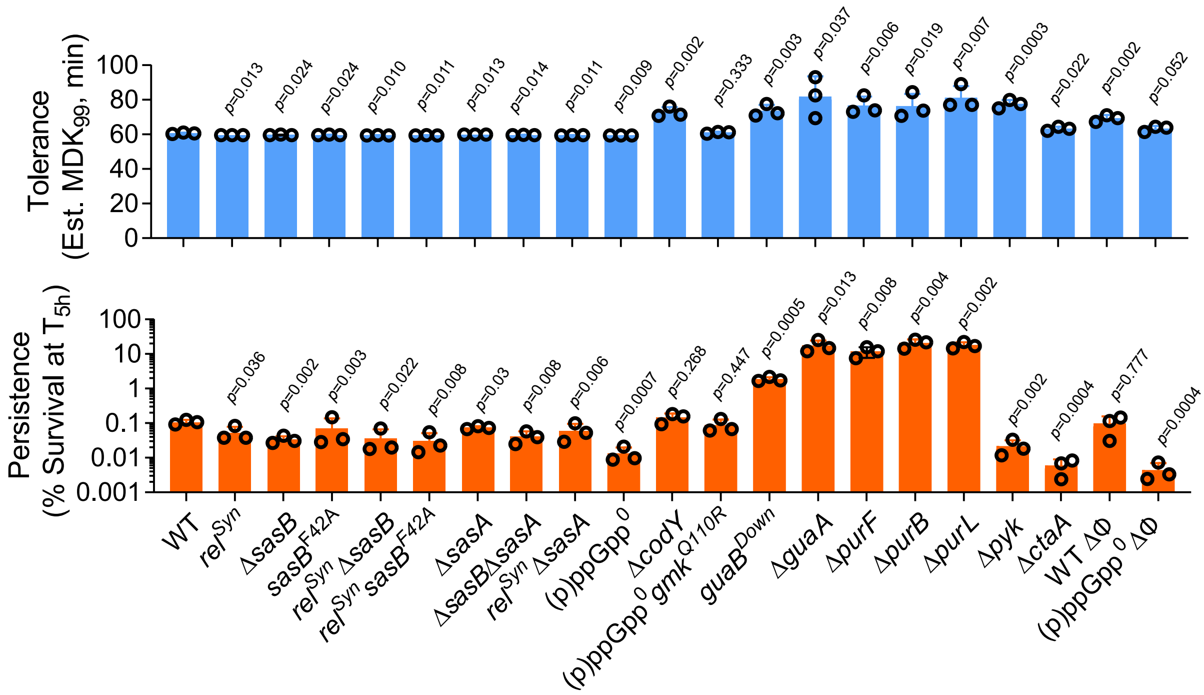

Supplemental Data Fig. 5. Population tolerance and persistence of exponentially growing wild type and (p)ppGpp or purine mutants. Bar graph of tolerance and persistence values of of wild type and (p)ppGpp or purine mutants. Tolerance was determined from the killing phase of the survival curve as estimated MDK_{99} (minimal duration of killing of 99% of population). Persistence was determined from the fraction of survivors in the antibiotic refractory phase (5h) in the survival curves from Fig 2b-c and Supplemental Data Figure 4.Values represent mean ± s.d., three biological replicates. *p* values were derived from unpaired two-tailed t-test between WT and mutant pairs. See also Supplemental Data Table 7 for a summary table of growth rates, tolerance and persistence of wild type and (p)ppGpp or purine mutants.

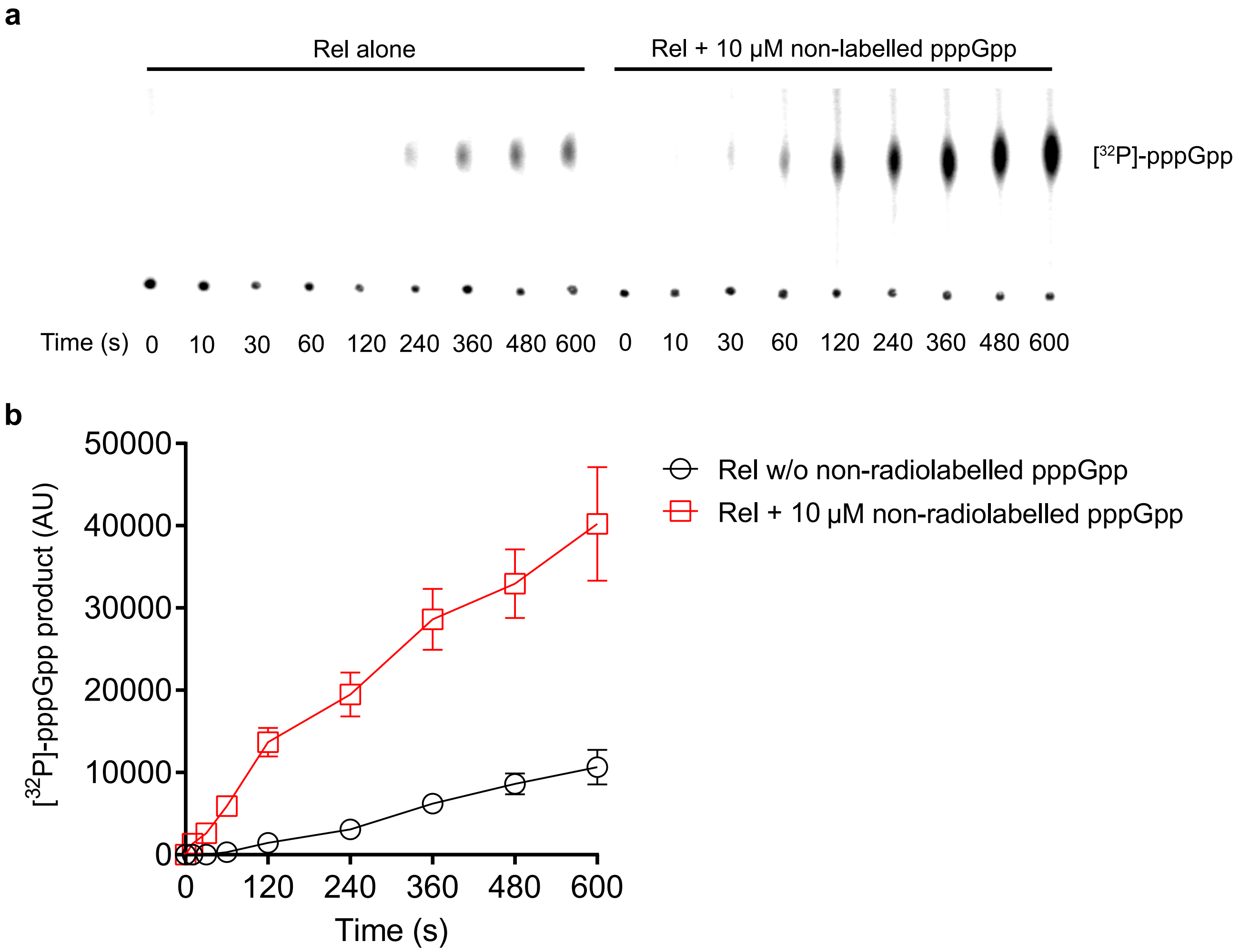

Supplemental Data Fig. 6. pppGpp stimulates pppGpp synthesis by Rel *in vitro*. (a) Representative thin layer chromatography (TLC) image of radiolabelled pppGpp synthesis by Rel in the absence or presence of 10 μM non-radiolabelled pppGpp in the absence of ribosomes. The reaction contains 236 nM *B. subtilis* Rel, 0.05 μM [α^{32}P] GTP, and 1 mM ATP in 20 mM Tris-Cl pH 7.5, 50 mM NaCl and 10 mM $MgCl_2$ at 37°C. Reaction is initiated by addition of ATP and devoid of manganese to avoid (p)ppGpp hydrolysis. (b) [^{32}P]-pppGpp synthesis over time by Rel with or without 10 μM non-radiolabelled pppGpp. Values represent mean ± s.d. from three biological replicates .

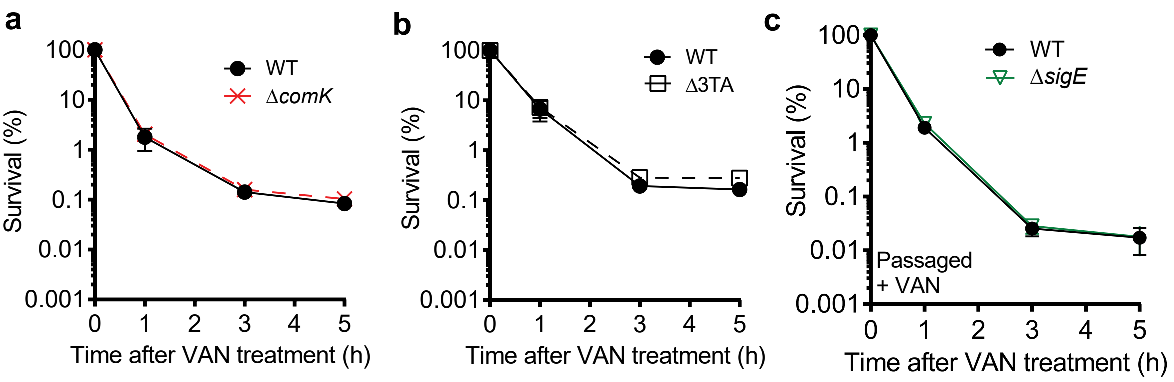

Supplemental Data Fig. 7. Disruption of competence, sporulation, or three known toxin-antitoxin systems in *B. subtilis* has little impact on (p)ppGpp-mediated persistence. Kill curves of (a) WT and a mutant lacking *comK* required for development of competence (Δ*comK*), (b) WT and a mutant lacking three known toxin-antitoxin systems toxins *ydcE*, *yonT* and *txpA* (Δ3TA), or (c) WT and a mutant lacking the sporulation sigma factor sigE (Δ*sigE).* Cells were grown to exponential phase and subjected to vancomycin (VAN) treatment for 5 h. Passaged+ VAN: Cells were grown to exponential phase and after one generation of serial passage before vancomycin treatment. Values represent mean and error bars represent s.e.m., three biological replicates .

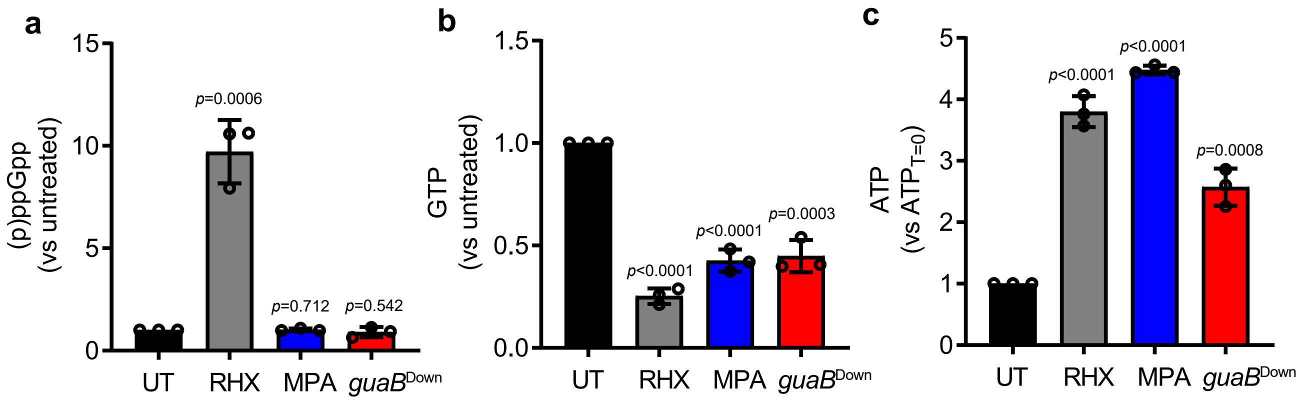

Supplemental Data Fig. 8. Repression of *guaB* expression (*guaB*↓) or activity reduces GTP but does not induce (p)ppGpp or deplete ATP. (a-c) Levels of (a) (p)ppGpp, (b) GTP, and (c) ATP in response to treatment with amino acid starvation inducer arginine hydroxamate (RHX), or GuaB inhibitor mycophenolic acid (MPA), or repression of *guaB* expression ($guaB^{Down}$) using IPTG-dependent promoter growing in the absence of IPTG. Nucleotide levels were normalized to their levels before treatment or repression of *guaB* expression. Values represent mean and error bars indicate s.d. from three biological replicates. *p* values were derived from unpaired two-tailed t-test between treated and untreated cells (UT).

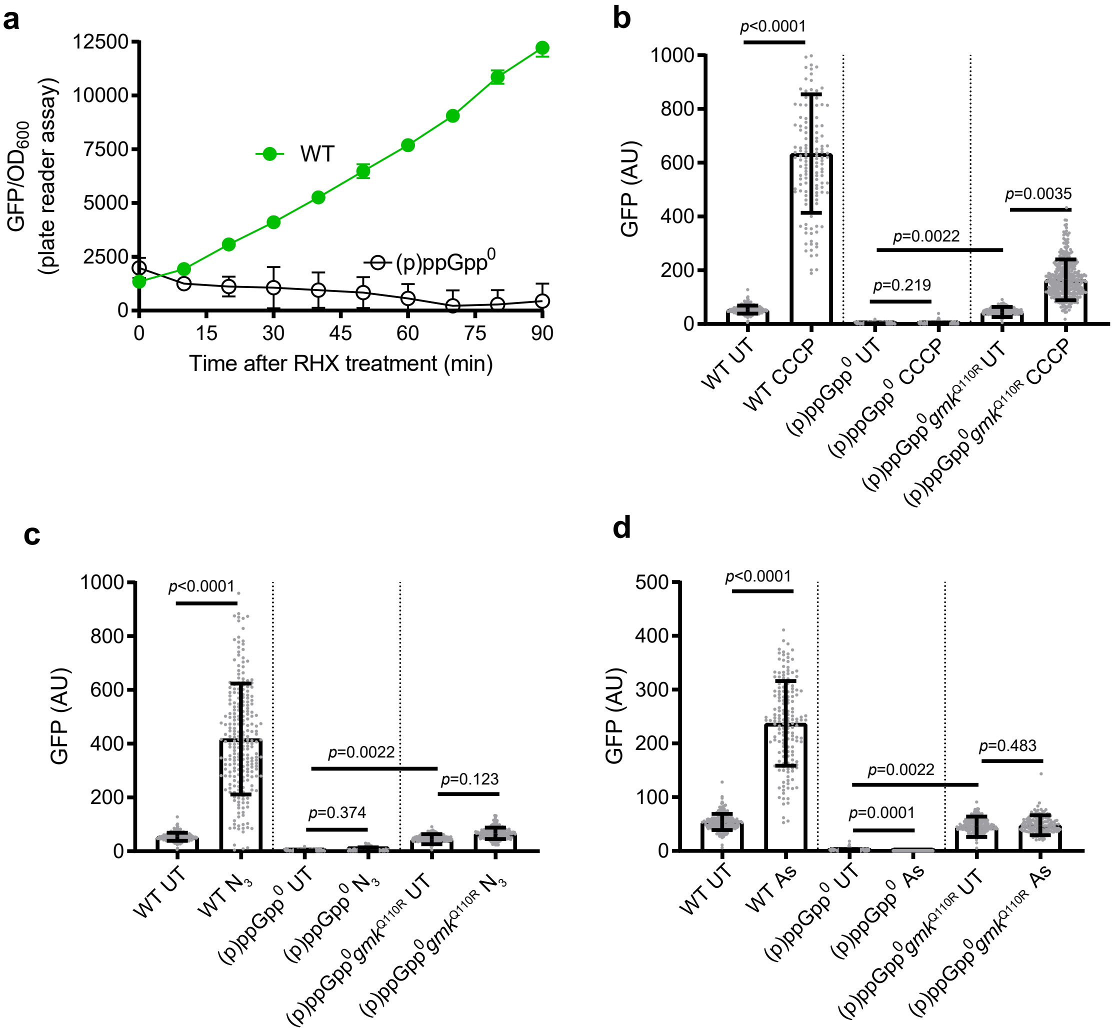

Supplemental Data Fig. 9. P_{lowGTP} response to amino acid or ATP starvation is abolished in the absence of (p)ppGpp. (a) Changes in P_{lowGTP} fluorescence in wild type (WT) or (p)ppGpp^0 in response to (p)ppGpp induction using arginine hydroxamate. (b-d) Microscopy measurement of P_{lowGTP} fluorescence in untreated (UT) wild type, (p)ppGpp^0, and (p)ppGpp^0 *gmk*^{Q110R} or after treatment to (b) 5 µM CCCP, (c) 4 mM sodium azide (N_3) or (d) 2.5 mM arsenate (As) for 30 min. ~200 cells each from three biological replicates. AU stands for arbitrary units. Values represent mean and error bars represent s.d. *p* values were derived from unpaired two-tailed t-test

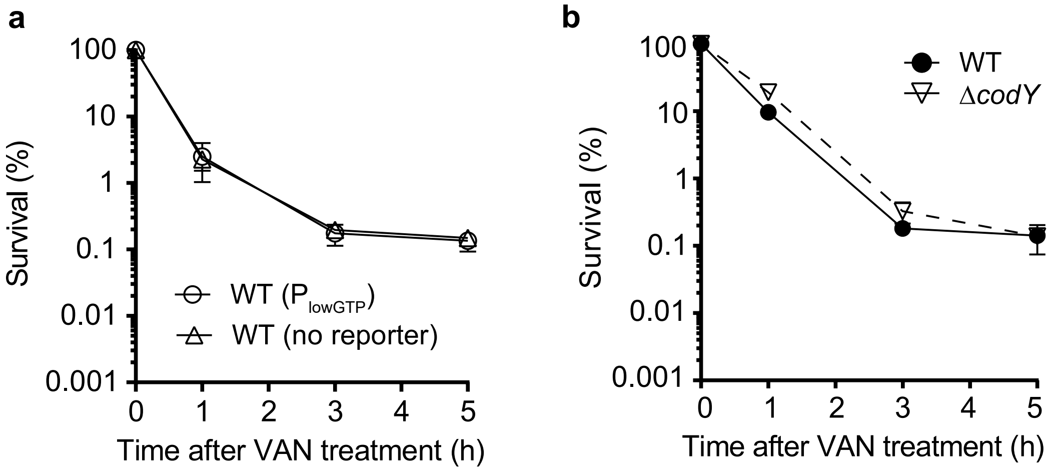

Supplemental Data Fig. 10. P_{lowGTP} reporter or CodY repressor do not affect persistence. (a) Vancomycin kill curves of wild type (WT) with or without the P_{lowGTP} reporter. (b) Vancomycin kill curves of WT and Δ*codY* mutant. Vancomycin was used at 20x MIC. Values represent mean and error bars represent s.d. from three biological replicates. The slight increase in persistence in Δ*codY* cells is likely due to the small amount of GTP reduction in the mutant, since we had previously observed that CodY is a positive regulator of GTP biosynthesis genes.

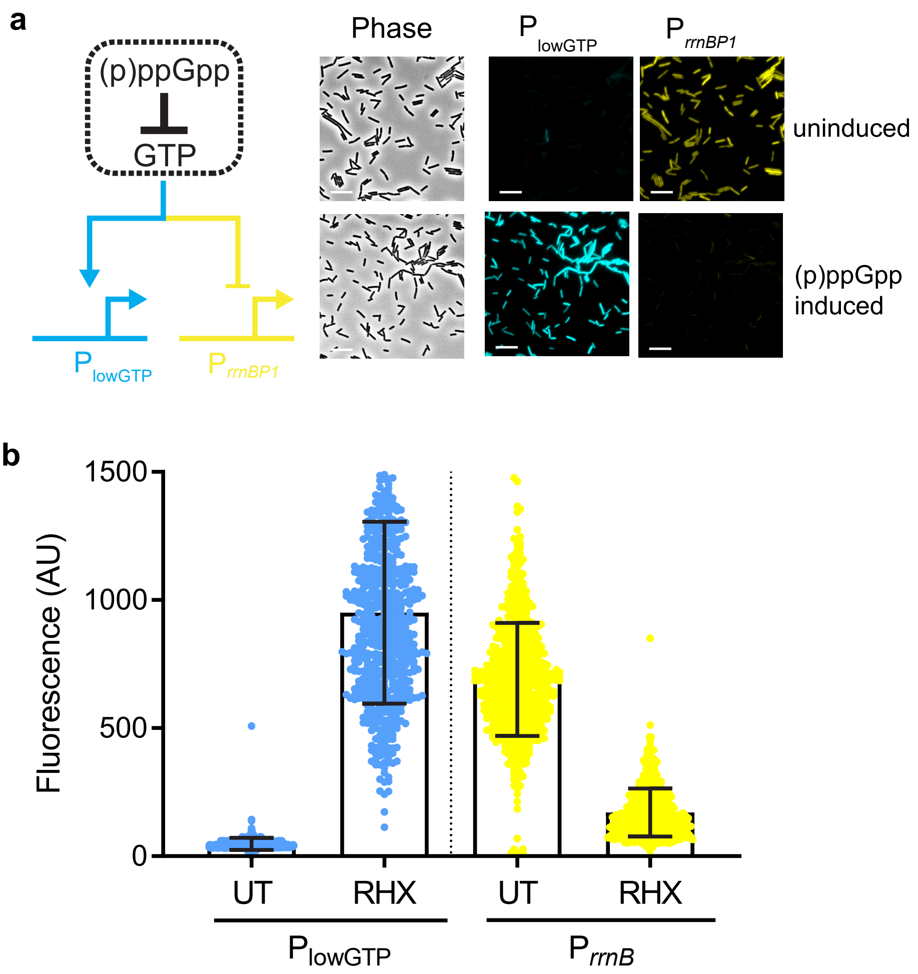

Supplemental Data Fig. 11. P_{lowGTP} fluorescence is reciprocal to that of high GTP reporter. a) Wild type containing both P_{lowGTP} (activated by low GTP) and P_{rrnBP1} reporter (activated by high GTP) were imaged before and after 60 min amino acids starvation by arginine hydroxymate treatment. Scale bar: 10 μm. b) Quantitation of data from a), ~600 cells each from three biological replicates. AU stands for arbitrary units. Values represent mean and error bars represent s.d.

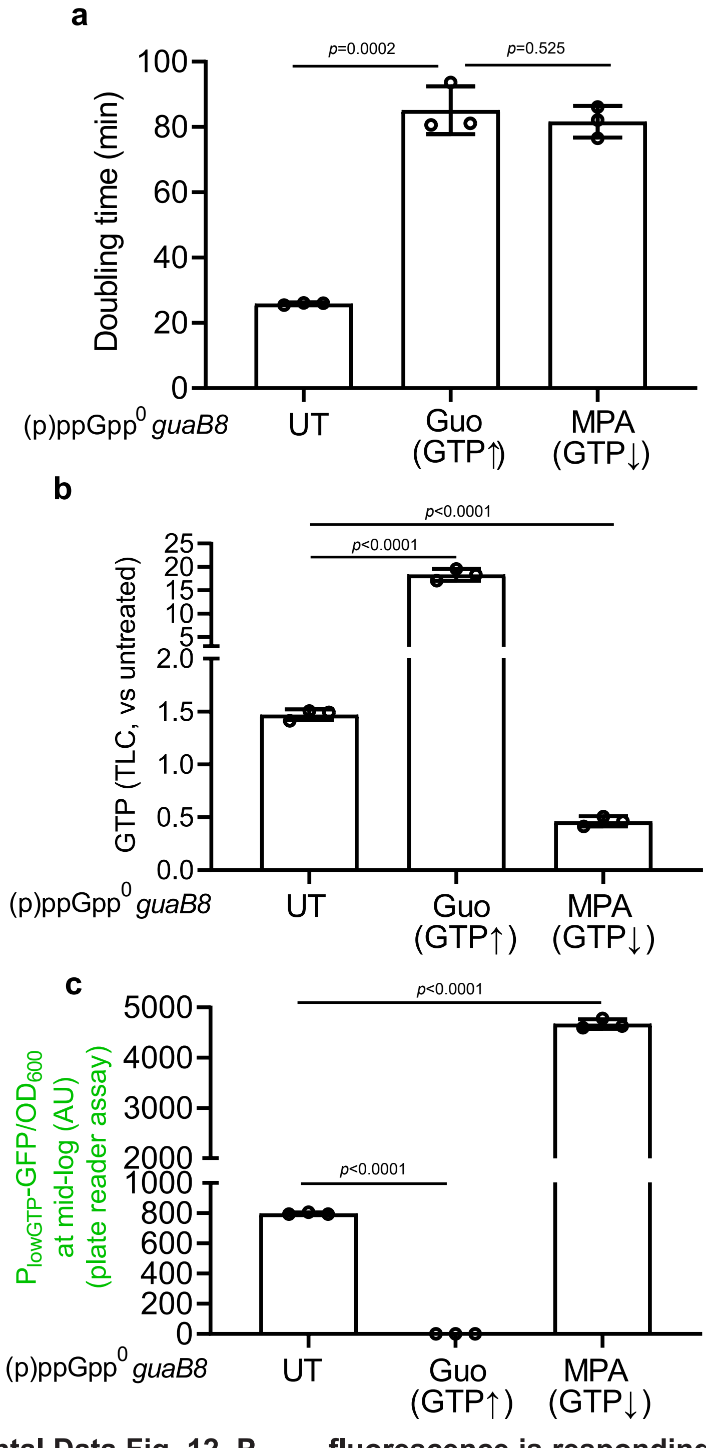

Supplemental Data Fig. 12. P_{lowGTP} fluorescence is responding to GTP rather than slow growth. Since wild type *B. subtilis* robustly regulates its GTP levels through (p)ppGpp synthesis, we used the (p)ppGpp^0 *guaB8* mutant[34] to chemically manipulate cellular GTP to achieve slow growth at either high or low GTP levels to test whether the P_{lowGTP} reporter responds to slow growth or GTP levels. a) Doubling time of (p)ppGpp^0 *guaB8* mutant treated with either Guanosine (Guo) or GuaB inhibitor mycophenolic acid (MPA) which leads to similar growth reduction. b) GTP levels in (p)ppGpp^0 *guaB8* mutant treated with either Guanosine (Guo) or GuaB inhibitor mycophenolic acid (MPA) which results in either GTP hyperaccumulation or GTP depletion. Nucleotide levels were measured by thin layer chromatography (TLC) and normalized to their levels before treatment (UT). c) P_{lowGTP} reporter fluorescence in (p)ppGpp^0 *guaB8* mutant treated with either Guanosine (Guo) or GuaB inhibitor mycophenolic acid (MPA). Despite both treatments led to similar slow growth, cells with high GTP have low reporter fluorescence, while cells with low GTP have high reporter fluorescence. This demonstrates that the reporter responds to changes in GTP rather than slow growth. Values represent mean and error bars represent s.d., three biological replicates. GFP signals were normalized to OD_{600}. *p* values were derived from unpaired two-tailed t-test.

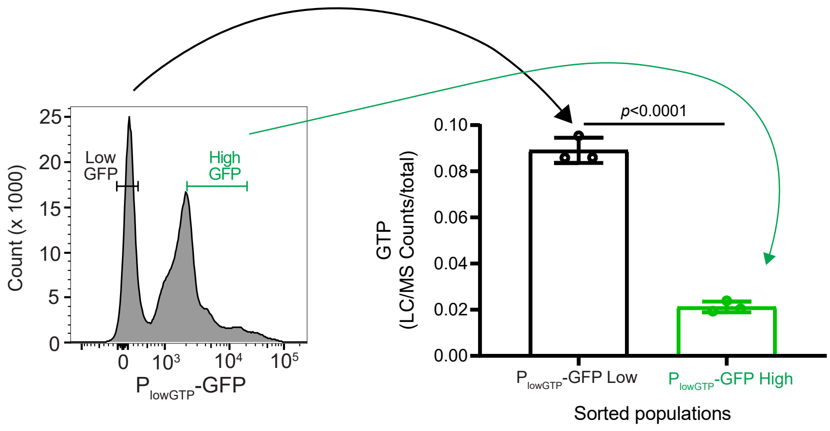

Supplemental Data Fig. 13. Cells with high P_{lowGTP} fluorescence have lower GTP. Log phase and stationary phase wild type cells containing P_{lowGTP} reporter were mixed in roughly equal numbers and FACS-sorted into low GFP and high GFP populations, then immediately subjected to metabolite extraction and LC-MS quantitation of GTP. Approximately 8 x 10^6 cells were sorted per sample per replicate. LC/MS counts were normalized to the internal standard of six most represented nucleotides detected in the sample. Values represent mean and error bars indicate s.d. from three biological replicates. *p* values were derived from unpaired two-tailed t-test.

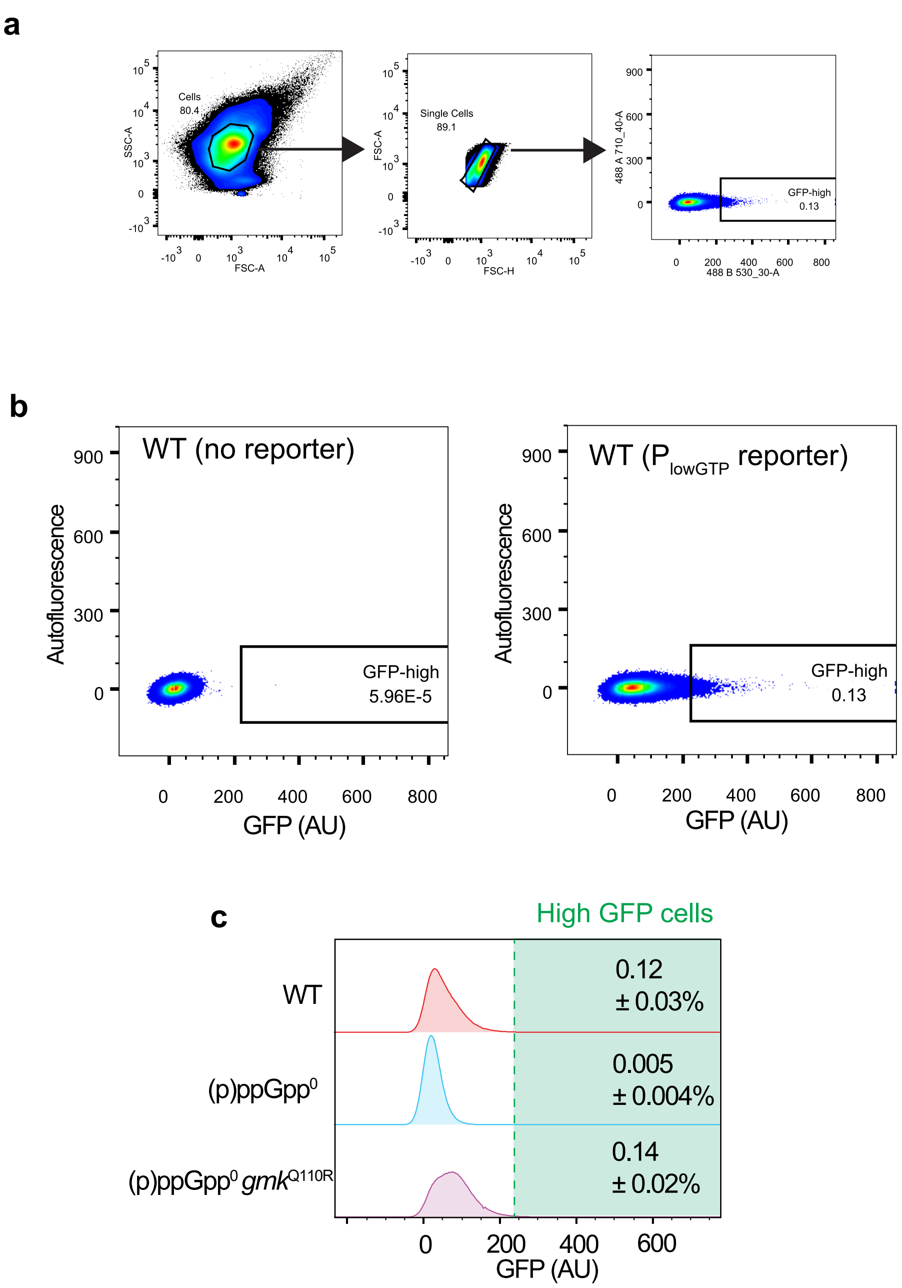

Supplemental Data Fig. 14. Flow cytometry analysis of populations containing the P_{lowGTP} reporter. (a) Gating strategy for flow cytometry or FACS analysis of cells with high P_{lowGTP} fluorescence. Cells within a narrow range of cell sizes (”Cells”) were gated, sub-gated to filter cell aggregates (”Single cells”), and analyzed for their fluorescence distribution or for cell sorting. SSC: side-scatter, FSC: forward-scatter, 488 A710_40: autofluorescence, 488 B530_30: GFP (P_{lowGTP} reporter) fluorescence. Numbers indicate the fraction (%) of GFP-high cells. ~$1.5x10^6$ cells in each sample. (b) Scatter plots of wild type (WT) populations without or with the P_{lowGTP} reporter. (c) Histogram of exponentially growing wild-type, (p)ppGpp^0, and (p)ppGpp^0*gmk*^{Q110R} populations containing the P_{lowGTP} reporter. The shaded area indicates the fraction (%, mean ± s.d.) of high P_{lowGTP} fluorescence cells. ~$1.5x10^6$ cells were analyzed per replicate, three biological replicates. The fluorescence cut-off was chosen based on FACS and antibiotic treatment experiment that these high fluorescence cells display persistence phenotype (see figure 3a-b).

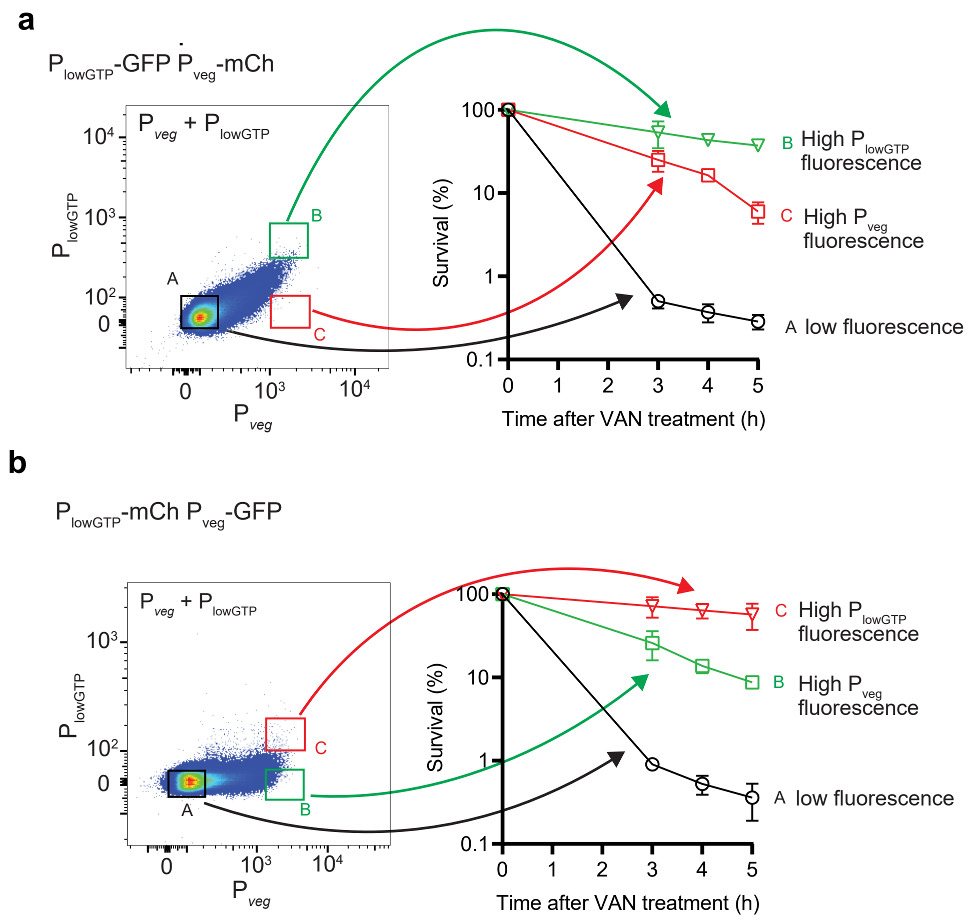

Supplemental Data Fig. 15. Cells with high P_{lowGTP} fluorescence display persistence phenotype. (a) Wild type containing both P_{lowGTP}-GFP and P_{veg}-mCherry reporters were grown to exponential phase and FACS-sorted into A: low P_{veg} and P_{lowGTP} fluorescence, B: high P_{lowGTP} fluorescence, and C: High P_{veg} fluorescence into tubes containing fresh growth media and vancomycin. Survival of sorted cells was monitored over time by serial dilution and plating on agar plates without antibiotics. Number of cells before treatment (T_0) were measured by the cell sorter. (b) Same experiment as (a) but with swapped fluorescent proteins (i.e. P_{lowGTP}-mCherry and P_{veg}-GFP reporters). Approximately 1 x 10^6 cells were sorted per sample per replicate. Values represent mean ± s.d., three biological replicates.

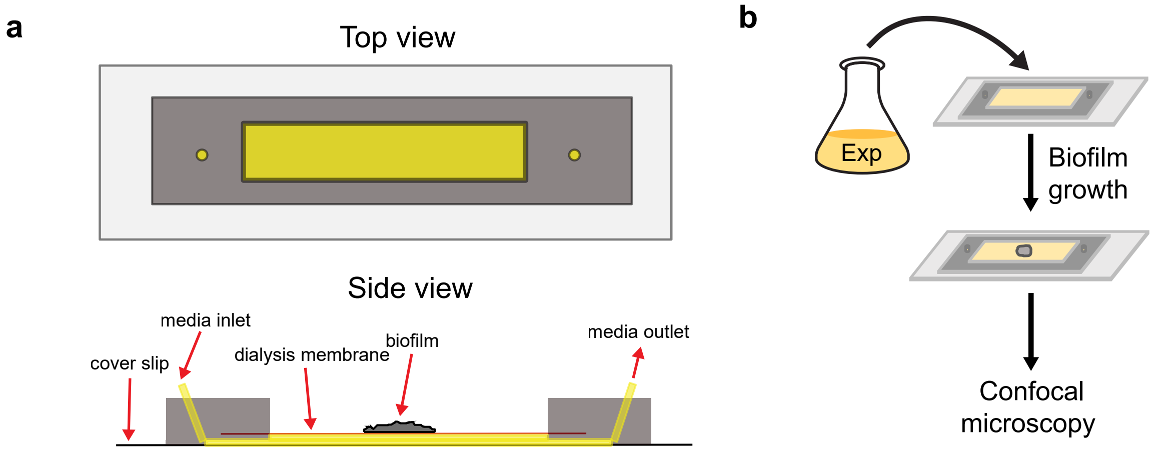

Supplemental Data Fig. 16. Growth of *B. subtilis* microfluidic biofilm. (a) Schematic of microfluidic biofilm device. The device is composed of a dialysis membrane fixed on a PDMS mold containing media inlet and outlet channels underneath. (b) Cells are inoculated on the membrane and grew into biofilm with non-disruptive media flow under the membrane. Grown biofilm was observed using confocal microscopy.

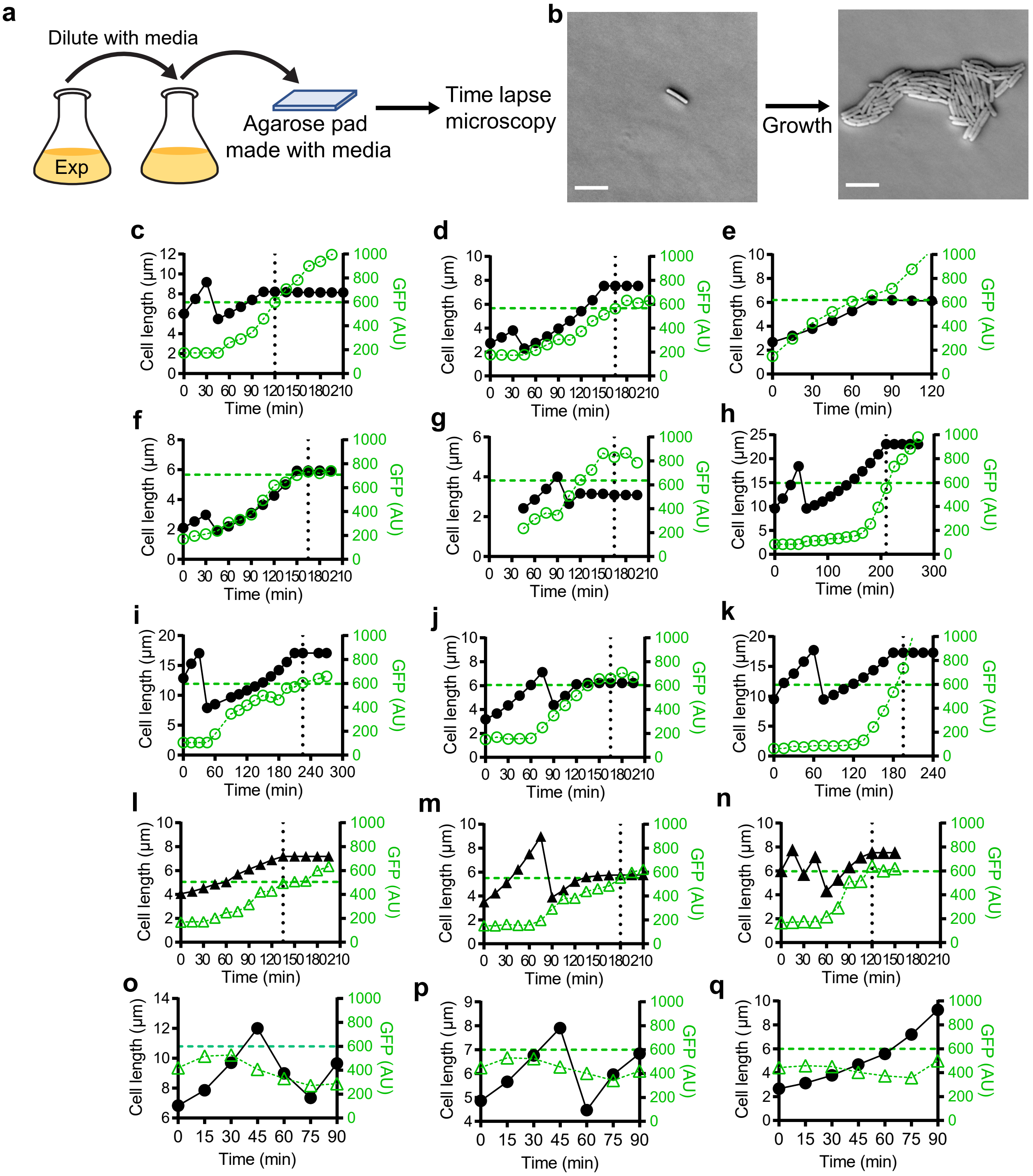

Supplemental Data Fig. 17. Spontaneous formation of low-GTP persisters. (a) Schematic of timelapse microscopy experiment to monitor growth and persister formation. Wild type or (p)ppGpp^0 (GTP↓) mutant containing the P_{lowGTP} reporter were grown in liquid media to exponential phase, then diluted and patched on agarose pads made with fresh growth media. Cell growth on agarose pad is monitored with timelapse microscopy. An example is provided in (b). Scale bar: 10 μm. (c-n) Single cell traces of spontaneous persistence entrance in (c-k) wild type and (l-n) (p)ppGpp^0 *gmk*^{Q110R} mutant. Changes in P_{lowGTP} reporter fluorescence (green) and cell length (black) are shown. The periodic drop in cell length in the traces are due to cell division. Horizontal green dash lines indicate the fluoresence threshold (~600 units in our setup) over which the cell enter presistence. (o-q) Single cell traces of wild type cells which kept growing when reporter fluorescence is near but below the threshold (e.g just below 600 units). This indicates that even if GFP is increasing in some cells to close the threshold, if GFP does not reach the threshold, cells do not enter persistence.

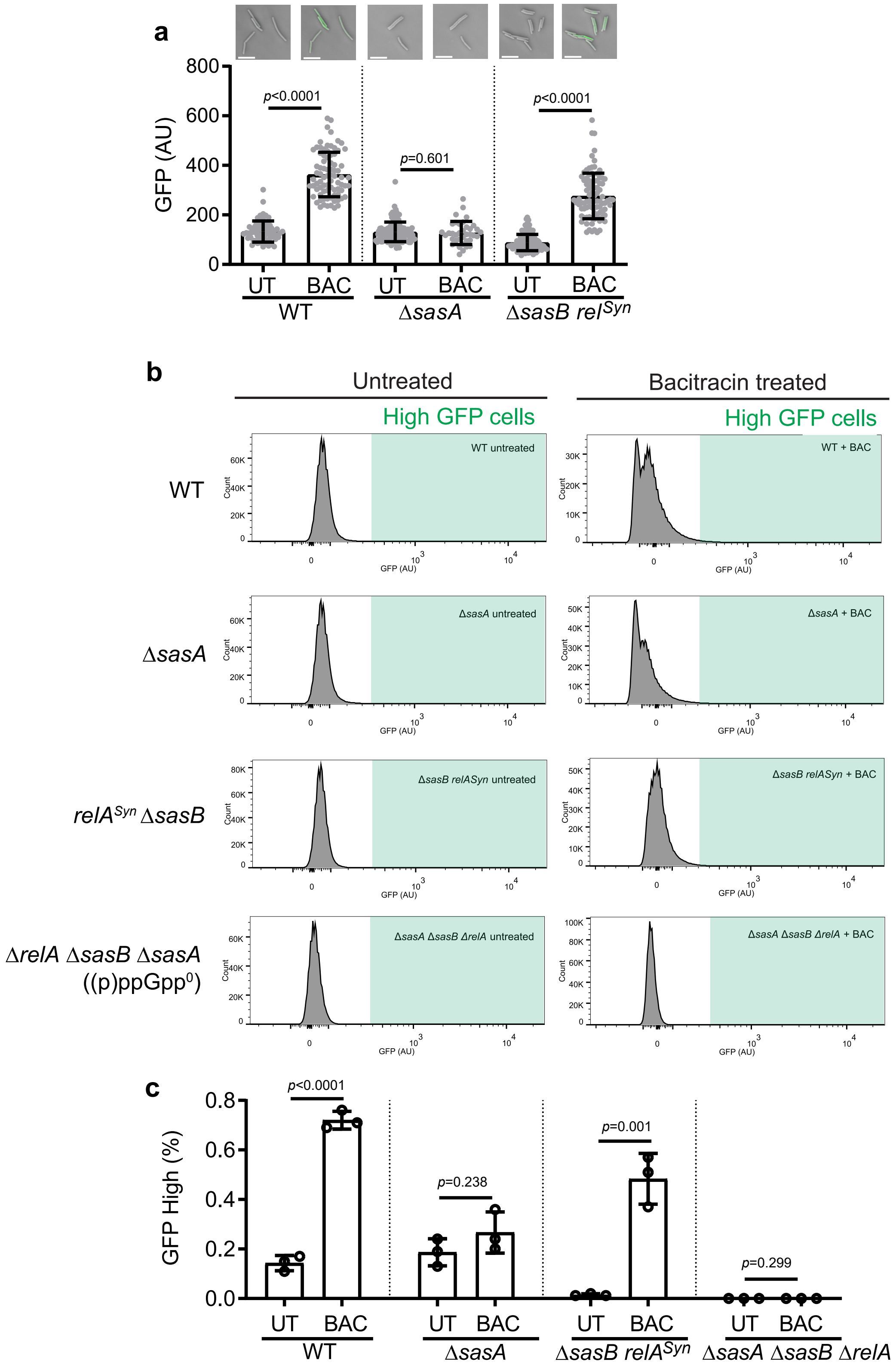

Supplemental Data Figure 18. Cell wall antibiotic treatment induces the formation of high P_{lowGTP} fluorescence cells. Exponentially growing wild type (WT), Δ*sasA,* Δ*sasB rel*^{Syn}, or Δ*sasA* Δ*sasB* Δ*relA* containing the P_{lowGTP} reporter were treated with 3x MIC bacitracin for 60 min followed by microscopy or flow cytometry analysis (~ 1x10^{6} cells each, three biological replicates). (a) Induction of high P_{lowGTP} fluoresence cells measured using microscopy (> 200 cells each, three biological replicates) UT: before treatment. Scale bar: 10 µm. (b) Induction of high P_{lowGTP} fluoresence cells measured using flow cytometry. Histograms showing the distribution of high P_{lowGTP} fluoresence cells. The shaded area indicates the fraction of high P_{lowGTP} fluorescence cells. (c) Quantitation of cells with high P_{lowGTP} fluorescence from b). UT: before treatment. BAC: after bacitracin treatment. For a) and b), values represent mean and error bars represent s.d. from three biological replicates. *p* values were derived from unpaired two-tailed t-test.

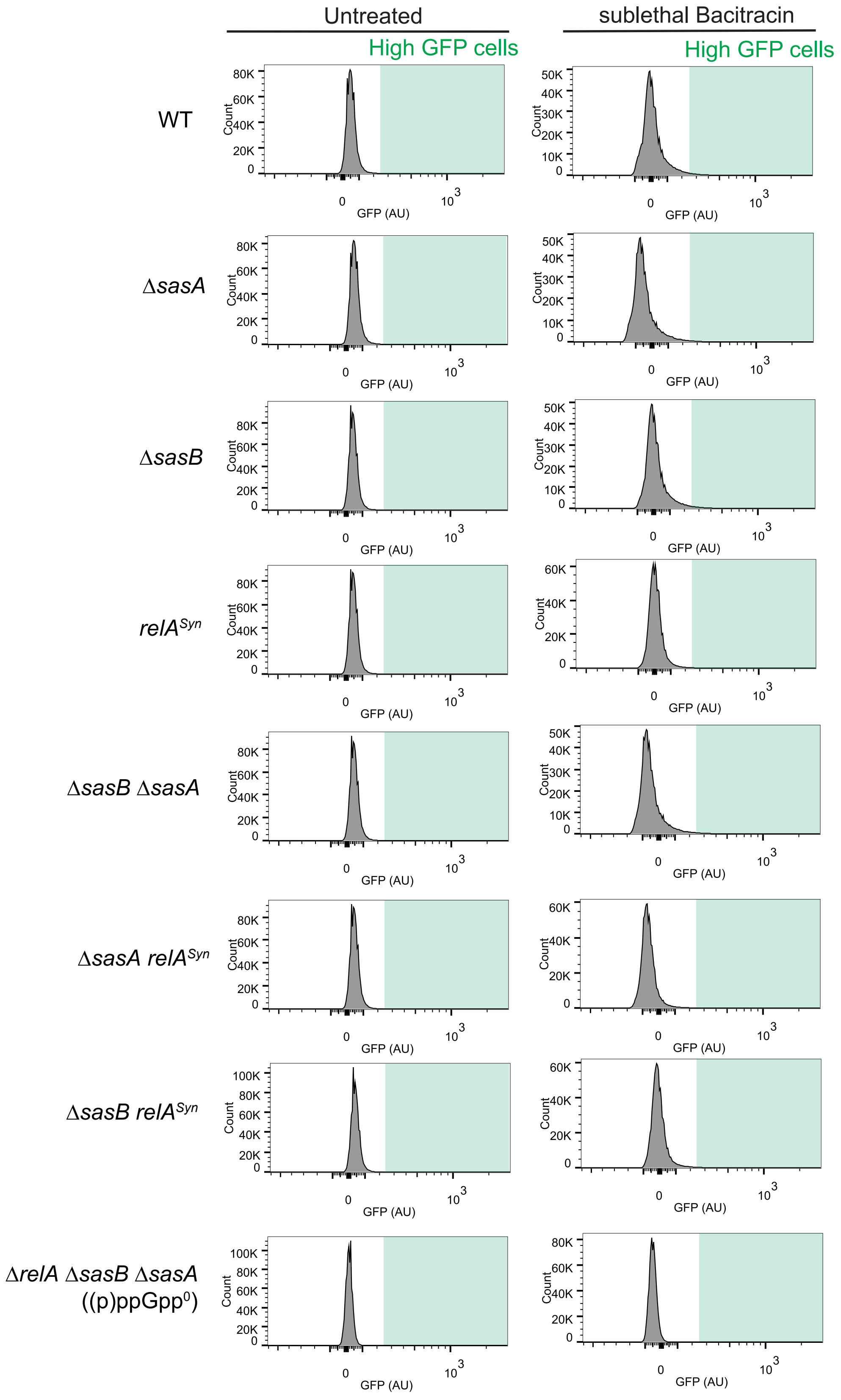

Supplemental Data Figure 19. Flow cytometry analysis of high P_{lowGTP} fluorescence cells before and after sublethal bacitracin treament. Exponentially growing wild type (WT) and (p)ppGpp mutants containing the P_{lowGTP} reporter were treated with 0.5 x MIC bacitracin for 30 min followed by flow cytometry analysis (~ $1x10^6$ cells each, three biological replicates). Histograms showing the distribution of high P_{lowGTP} fluoresence cells after subtraction of autofluorescence. The shaded area indicates the fraction of high P_{lowGTP} fluorescence cells. Quantitation of cells with high P_{lowGTP} fluorescence is shown in figure 4e.

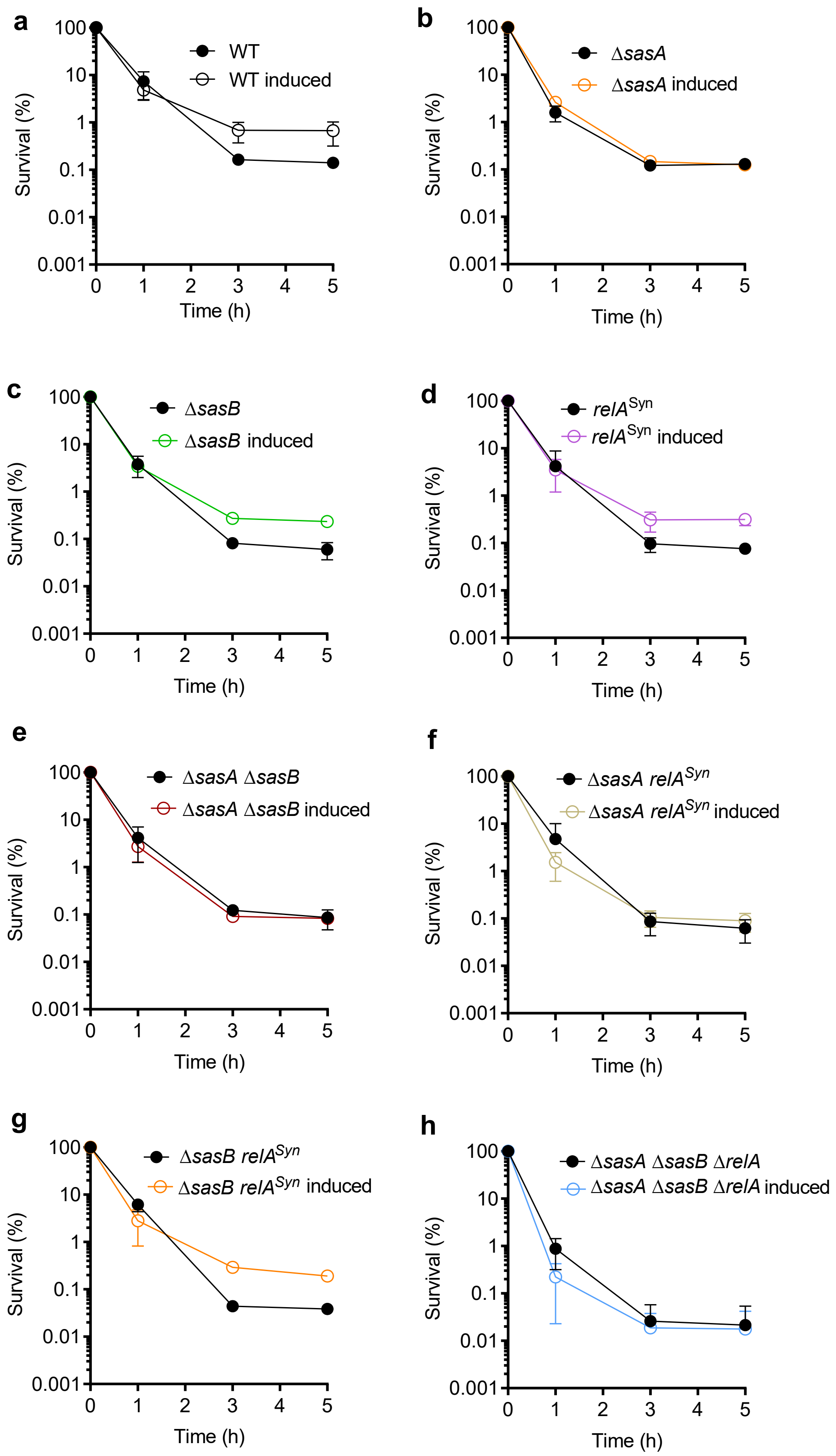

Supplemental Data Fig. 20. Persistence induction by sublethal cell wall antibiotic. (a-h) Survival of WT or (p)ppGpp mutants with or without 30 min pretreatment with sublethal (0.5x MIC) bacitracin, followed by lethal (3x MIC) bacitracin treatment for up to 5 h. Values represent mean and error bars represent s.d. from three biological replicates.

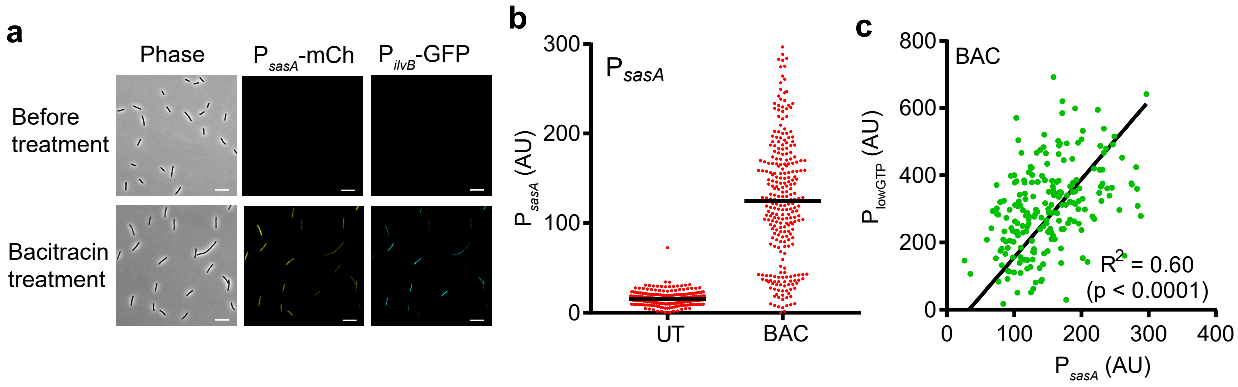

Supplemental Data Figure 21. Induction of SasA expression by cell wall antibiotic. (a) Representative image of cells containing both P_{lowGTP} and P_{sasA} reporters before and after treatment of sublethal (0.5x MIC) bacitracin for 1h. Scale bar: 10 μm. b) P_{sasA} fluorescence before and after bacitracin treatment from (a). (c) Correlation of P_{sasA} and P_{lowGTP} fluoresence before and after bacitracin treatment from (a). Line indicates linear regression. n > 200 cells, three biological replicates. R square was estimated using linear regression.

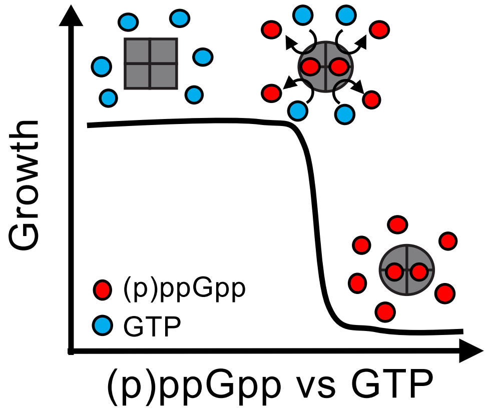

Supplemental Data Fig. 22. Switch-like persister formation by enzyme cooperativity. Our data suggest that self-amplification of (p)ppGpp, such as through SasB and Rel, is necessary for spontaneous persister formation from a small input signal, such as noises in (p)ppGpp synthesis. While (p)ppGpp accumulation in spontaneous persistence can be purely stochastic, we cannot rule out the possibility that (p)ppGpp could be triggered by transient stresses imposed on individual cells, such as localized nutrient fluctuation, or other stresses. Whether these potential mechanisms are in play remains to be explored.

Supplemental Data Table 1. Bacterial strains used in this study

	Genotype	Source
JDW2144	3610 *comI*^{Q21L} Prototroph (pBS32 minus)	Daniel Kearns
JDW2230	JDW2144 Δ*sasB* Δ*sasA* Δ*rel*::mls	This work
JDW2234	JDW2144 Δ*pyk*	Lab collection
JDW2528	JDW2144 Δ*sasB* Δ*sasA* Δ*rel*::mls *gmk*^{Q110R}	This work
JDW2721	JDW2144 *rel*^{D264G}	This work
JDW2901	3610 Δ*zpdN* ΔSPβ ΔPBSX Δ*comI*	Daniel Kearns
JDW2911	JDW2144 Δ*ydcE*::*erm* Δ*yonT* Δ*txpA*	This work
JDW2914	JDW2144 Δ*codY*	This work
JDW2921	JDW2144 *amyE*::P_{lowGTP}-GFPmut2^{A206K}	This work
JDW2923	JDW2144 Δ*sasB* *amyE*::P_{lowGTP}-GFPmut2^{A206K}	This work
JDW2927	JDW2144 Δ*sasB* Δ*sasA* Δ*rel::mls* *amyE*::P_{lowGTP}-GFPmut2^{A206K}	This work
JDW2935	JDW2144 *rel*^{D264G} *amyE*::P_{lowGTP}-GFPmut2^{A206K}	This work
JDW2937	JDW2144 Δ*sasA* *amyE*::P_{lowGTP}-GFPmut2^{A206K}	This work
JDW2939	JDW2144 Δ*sasB* Δ*sasA* *amyE*::P_{lowGTP}-GFPmut2^{A206K}	This work
JDW2941	JDW2144 *rel*^{D264G} Δ*sasB* *amyE*::P_{lowGTP}-GFPmut2^{A206K}	This work
JDW2947	JDW2144 Δ*sasA* *rel*^{D264G} *amyE*::P_{lowGTP}-GFPmut2^{A206K}	This work
JDW2949	JDW2144 *sasB*^{F42A} *amyE*::P_{lowGTP}-GFPmut2^{A206K}	This work
JDW2951	JDW2144 *sasB*^{F42A} *rel*^{D264G} *amyE*::P_{lowGTP}-GFPmut2^{A206K}	This work
JDW2953	JDW2144 Δ*sasB* Δ*sasA* Δ*rel* *gmk*^{Q110R} *amyE*::P_{lowGTP}-GFPmut2^{A206K}	This work
JDW2963	JDW2144 *lacA*:: P_{rrnB}-GFPns *amyE*::P_{lowGTP}-mCherry	This work
JDW3021	JDW2901 Δ*sasB* Δ*sasA* Δ*rel*::mls	This work
JDW3031	JDW2144 *guaB*::pJW305 (*guaB*'-*lacZ* *erm* P_{spac}-*guaB*)	This work
JDW3529	YB886 Δ*sasB* Δ*sasA::kan* Δ*rel::mls* *guaB*(1276G-A) *amyE*:: P_{lowGTP}-GFPmut2^{A206K}	This work
JDW3935	JDW2901 Δ*sigE::erm*	This work
JDW4017	JDW2901 Δ*sasA* Δ*sasB* Δ*rel::mls* *amyE::*P_{spank}-*sasA*	Lab collection
JDW4019	JDW2901 Δ*sasA* Δ*sasB* Δ*rel::mls* *amyE::*P_{spank}-*sasA*^{D87G}	Lab collection
JDW3989	JDW2144 Δ*guaA::kan*	This work
JDW3990	JDW2144 Δ*purB::kan*	This work
JDW3991	JDW2144 Δ*purL::kan*	This work
JDW3992	JDW2144 Δ*purF::kan*	This work
JDW3993	JDW2144 *amyE::* P_{lowGTP}-GFPmut2^{A206K} lacA::P_{veg}-mCherry	This work
JDW4195	JDW2144 *amyE::* P_{lowGTP}-mCherry lacA::P_{veg}-GFPmut2^{A206K}	This work
JDW4421	JDW2144 Δ*ctaA::kan*	This work
JDW3365	JDW2144 *amyE*::P_{ilvB}-GFPmut2^{A206K} *lacA*::P_{ywaC}-mCherry	This work

Supplemental Data Table 2. Plasmids used in this study

Name	Genotype	Source
pDR110	*amyE*::P_{spank} *amp spc*	David Rudner
pDR244	*cre* + Ts origin	BGSC
pJW239	pEX44/Δ*sasB amp cat*	Lab stock
pJW299	pEX44/I-SceI site *amp cat*	Lab stock
pJW300	pJW239/Δ*sasB* I-SceI site *amp cat*	Lab stock
pJW305	pMUTIN4/ P_{spac}-*guaB'-lacZ erm*	Lab stock
pJW306	pJW299/Δ*sasA* I-SceI site *amp cat*	Lab stock
pJW370	pJW299/*sasB* I-SceI site *amp cat*	Lab stock
pJW371	pJW299/rel^{D264G} I-SceI site *amp cat*	Lab stock
pJW562	$sasB^{F42A}$ I-SceI site *amp cat*	This work
pSS4332	*oriU* P_{amy}-I-sceI *kan*	Scott Stibitz
pJW753	pE-SUMO-*rel(Bs) kan*	This work

Supplemental Data Table 3. Primers used in this study

Name	Purpose	Sequence (5' → 3')
oJW358	Verify Δ*sasB* (F)	ATGTATGGCCGGAACTGAAG
oJW359	Verify Δ*sasB* (R)	CGGTCGCTGTATCTGTGAAA
oJW418	Verify Δ*relA* (F)	TTTGGCTATTTGAACTTCTGCTC
oJW419	Verify Δ*relA* (R)	TTCGTCCACTGTAACGCTTG
oJW904	Verify Δ*sasA* (F)	CGTCCTCATACGTTAACCGC
oJW905	Verify Δ*sasA* (R)	GGGCTATCAAAAGGACTTTACCG
oJW902	Amplify *rel::mls* (F)	AAAGAGGCGCTTTTGACGTG
oJW903	Amplify *rel::mls* (R)	TTGTTGACCCGGGACATGGA
oJW1935	Amplify P_{lowGTP} (F)	CAGACTCAATCCATATACGAAC
oJW1936	Amplify P_{lowGTP} (R)	TTTAGTTCCTCCTTTTGGATTTTC
oJW1995	Amplify *gfp* (F)	ATGAGTAAAGGAGAAGAACTTTTC
oJW1996	Amplify *gfp* (R)	TTATTTGTATAGTTCATCCATGC
oJW2020	Amplify *gfpns* (R)	TTAAGCGCTATCAGCATAATTTTCGCTATAATTTTCATCATTTGCAGCTTTGTATAGTTCATCCATGC
oJW2083	Amplify P_{rrnB} (F)	TTTAAAATTTCCTCCTTATAAATGG
oJW2084	Amplify P_{rrnB} (R)	GCTGCCGCTGTCATAATG
oJW1990	Amplify *lacA-up* (F)	TCGATATGGGCGGATTCTTTTTC
oJW2414	Amplify *lacA-up* (R)	TCAGCGTTTCTTTGGAATATGTCCTG
oJW2413	Amplify *lacA-dn* (F)	ATGCTGGCAGATATGAAAGTCAGAAGA
oJW2082	Amplify *lacA-dn* (R)	TCTCCTCCTTGTTCTCTTAGC
oJW2133	Amplify *lox-ermR-lox* (F)	GAGGGAGGAAAGGCAGGATA
oJW2134	Amplify *lox-ermR-lox* (R)	CGCCGTATCTGTGCTCTCTC
oJW2309	$sasB^{F42A}$ substitution in pJW370 (F)	CCATTCACCGATCGAAGCTGTGACCGGACGCGTC
oJW2310	$sasB^{F42A}$ substitution in pJW370 (R)	GACGCGTCCGGTCACAGCTTCGATCGGTGAATGG
oJW3099	Amplify P_{sasA} (F)	GGTTGTCGGCTTGGTTATTACC
oJW3079	Amplify P_{sasA} (R)	GTTCGTCATCTCCTTTAACGGAAC
oJW2805	Amplify *mcherry* (F)	ATGAGCAAAGGAGAAGAAGATAACATG
oJW2806	Amplify *mcherry* (R)	TTATTTGTAAAGCTCATCCATTCCGC
oJW3928	Amplify P_{veg} (F)	GGAGTTCTGAGAATTGGTATGCC
oJW3196	Amplify *rel* (F)	GTGGGTCTCTAGGTATGGCGAACGAACAAGTATTGACTGC
oJW3197	Amplify *rel* (R)	GTGGGTCTCTATTAGTTCATGACGCGGCGCACAGAA
oJW3194	Amplify pE-SUMO vector (F)	GCGGGTCTCAACCTCCAATCTGTTCGCGGTGAGCC
oJW3195	Amplify pE-SUMO vector (R)	GCGGGTCTCATAATCGAGCACCACCACCACCACCA

Supplemental Data Table 4. Oligonucleotide fragments used in this study

Name	Sequence (5’ → 3’)
P_{rrnBP1} fragment	GCTGCCGCTGTCATAATGGCAATTTCAATAAGGCGAACCAGTTGCTTAGATTGATTCATATCTATCTCCTTCCATTGACAGA<br>CAACCAAAAGGACAACAAAAAAGCAGCACGCGGAAGGATGAGAAAAGTTCCACGCACTGCTTTACGATCTGCAATTCATT<br>TGACTTTGCCACATCCCTACGCCAGCGTTAACTAACAGGTTCAAAGGGTCAGAACGAAACCGCTCACTCTCAGCTTTAAT<br>GCTCCCCTTGTGGTCATCAGTATTTAGTTCGTTTCTACTATACAAGAAAACGAAAAAAACAACAAGATCACATGACTGATGT<br>ATATGTTCTTTTAAGAAACTTATATGATACACACGCTTTAGAAATCATGGCGAGGATTATAGTTTATTTGTTTTATAGATTTTTTT<br>TAAAAAACTATTGCAATAAATAAATACAGGTGTTATATTATTAAACGTCGCTGATGCACAGCGGACACAAACTAGATGCTTCT<br>GCTTCATTGAGAAGTAACGAAATGATCTTTGAAAACTAAACAAGACAAAACGTACCTGTTAATTCAGTTTTTAAAAATCGCA<br>CTGCGATGTGCGTAGTCAGTCAAACTAGGGCCTGCACGACGCAGGTCACACAGGTGTCGCCGCAGGATGCGGTGAACT<br>TAACCTGTGATCCATTTATAAGGAGGAAATTTTAAA
P_{lowGTP} fragment	CAGACTCAATCCATATACGAACCATATCGTGTTTATATAAAATTAAATAATTCTGGTGTATTCTTGATAGATTAATAAAAAAATTA<br>TAAAAACTTTTAACATTTGCAATTCCTTTTGTACCAATAATGAAAGCtTATACAATATAGATTGATTAATCAAAATTGTCTAATAA<br>TTTTAAAAAATGCTGTTGACACTGCGTCCAAAGCGGCGTAATATGAGTTCAACAAAAGATAAATGCAAGCTTCACAAGCGA<br>AAATCATCGCAGTATGATTCTAAAAAAATGAAAAACAAACGACCTTCTTGAACAGCTGGAAAGCCGTTCCGAAGGCTGAAT<br>ATGAAAAGCGCAGATGAGGATAAGTAGCCTTGATAAAGTTTTCCACAGAGAACCGGGTTAGCTGAGAACCGGCGAAGCTT<br>TACAAGGTGAACTCGCCTCAGAGTGCCAGTCTGAAATGACAGTAGGACTTGGCCGGGTGAACTTGATTCACTCGTTACTA<br>AAGCGGATAGAAATATCCATGAGACGGCCGATTAACAGGCCGTAAACAAGGGTGGTAATTGAATAATCAGCTATCTAGCTA<br>ATGAAAAGATGATCTTTAAAGGATGAAAATCCAAAAGGAGGAACTAAA
P_{sasA} fragment	GGTTGTCGGCTTGGTTATTACCGGTGCCGCAAGCCCATGGCTGTTTGTCGTCTTTTTGAGCGTGCCTAAGCCGGTTCAG<br>GCAGTGAAGGGCTTCGTCCAGAACGAAATGCCGATGAATATGATTGTCGCAATGAAATCAACAGCCCAAACAAATACATTT<br>TTCGGATTCCTGCTCTCGATCGGATTATTGATCAGCTATTTCCGATAATAAAAAAGACCGCTCGTTTCATGCGGTCTTTTTTT<br>GTTACAATCGACCGCATTTTGTAAAAAAATTCATAGAACCTTGCAGCAGACAGGGACGTCTAGTACATGGACAGCGGATAA<br>AGTTCCGTTAAAGGAGATGACGAAC
P_{veg} fragment	GGAGTTCTGAGAATTGGTATGCCTTATAAGTCCAATTTTGTCAAAATAATTTTATTGACAACGTCTTATTAACGTTGATACCG<br>GTTAAATTTTATTTGACAAAAATGGGCTCGTGTTGTACAATAAATGT

Supplemental Data Table 5. Summary data of resistance, tolerance and persistence phenotypes to vancomycin treatment in starvation-induced wild type and (p)ppGpp^0 mutant.

Resistance, tolerance and persistence in starvation induced *B. subtilis* wild type and (p)ppGpp^0 mutant						
	Resistance to VAN[a] (MIC in µg/mL)		Tolerance to VAN[b] (Est. MDK99, min)		Persistence to VAN[b] (% survival at T5h)	
Genotype	MIC	SD	Mean	SD	Mean	SD
WT no starvation	0.2	0.04	60.63	0.43	0.11	0.02
WT RHX	0.2	0.04	71.68	13.65	55.76	5.10
WT CCCP	0.2	0.04	68.29	3.60	48.94	9.93
WT Stationary phase	0.2	0.04	66.11	0.99	48.51	4.83
WT As	0.2	0.04	91.17	16.88	9.46	3.62
WT RHX then into rich media	0.2	0.04	70.89	4.59	59.66	12.67
(p)ppGpp^0 no starvation	0.2	0.04	59.47	0.02	0.01	0.00
(p)ppGpp^0 *sasA* overexpression	0.2	0.04	63.65	0.98	51.88	5.07
(p)ppGpp^0 *sasA*^{Syn} overexpression	0.2	0.04	59.55	0.01	0.01	0.00
(p)ppGpp^0 RHX	0.2	0.04	178.84	0.45	< 0.01	0.01
(p)ppGpp^0 CCCP	0.2	0.04	298.57	1.17	< 0.51	0.40

[a]Resistance data was borrowed from supplemental data figure 1.

[b]Tolerance and persistence were measured from Extended data figure 2a and 2b and figure 1c.

Supplemental Data Table 6. Summary table of spontaneous persistence in wild type and (p)ppGpp synthetase mutants

**1st passage**	Survival (%) at 5h after VAN treatment			
Genotype	Mean	Lower 95%CI	Upper 95% CI	p value (vs WT)
WT	0.136	0.109	0.166	
relA ^{Syn}	0.053	0.016	0.151	0.0040
Δ*sasB*	0.034	0.018	0.060	0.0003
sasB ^{F42A}	0.070	0.060	0.081	0.0017
relA ^{Syn} Δ*sasB*	0.036	0.015	0.078	0.0007
relA ^{Syn} *sasB* ^{F42A}	0.031	0.005	0.043	0.0001
Δ*sasA*	0.074	0.058	0.094	0.0023
Δ*sasB* Δ*sasA*	0.041	0.013	0.112	0.0018
relA ^{Syn} Δ*sasA*	0.060	0.012	0.245	0.0137
(p)ppGpp^{0}	0.013	0.006	0.026	<0.0001

**2nd passage (1/100 of 1st)**	Survival (%) at 5h after VAN treatment			
Genotype	Mean	Lower 95%CI	Upper 95% CI	p value (vs WT)
WT	0.050	0.024	0.083	
relA ^{Syn}	0.012	0.006	0.022	0.0150
Δ*sasB*	0.012	0.003	0.042	0.0162
sasB ^{F42A}	0.017	0.005	0.050	0.0300
relA ^{Syn} Δ*sasB*	0.006	0.000	0.052	0.0081
relA ^{Syn} *sasB* ^{F42A}	0.007	0.001	0.037	0.0090
Δ*sasA*	0.019	0.002	0.056	0.0260
Δ*sasB* Δ*sasA*	0.011	0.001	0.078	0.0137
relA ^{Syn} Δ*sasA*	0.015	0.008	0.025	0.0203
(p)ppGpp^{0}	0.002	0.001	0.003	0.0047

*Data was obtained from supplemental data figure 4. Statistical tests were performed between WT and mutant (Student's t test). Confidence intervals (CI) were calculated based on binomial distribution.

Supplemental Data Table 7. Summary table of growth rates, tolerance and persistence of wild type and (p)ppGpp or purine mutants.

Growth rates, tolerance and persistence in $S7_{50}$ + Cas media in *B. subtilis* wild type and mutants						
	Doubling times (min)[a]		Tolerance (Est. MDK99, min)[b]		Persistence (% survival at T5h)[c]	
Genotype	Mean	90% CI	Mean	SD	Mean	SD
WT	26.8	±0.268	60.63	0.43	0.11	0.016
rel ^{Syn}	23.5	±0.235	59.59	0.04	0.05	0.007
Δ*sasB*	26.8	±0.268	59.67	0.19	0.03	0.000
sasB ^{F42A}	26.8	±0.268	59.65	0.23	0.07	0.002
rel ^{Syn} Δ*sasB*	24.10	±0.241	59.49	0.01	0.04	0.008
rel ^{Syn} *sasB* ^{F42A}	25.30	±0.253	59.52	0.08	0.03	0.002
Δ*sasA*	29.30	±0.293	59.94	0.03	0.07	0.002
Δ*sasB* Δ*sasA*	26.80	±0.268	59.59	0.09	0.04	0.002
rel ^{Syn} Δ*sasA*	22.40	±0.224	59.52	0.04	0.06	0.037
(p)ppGpp^0	24.10	±0.241	59.47	0.02	0.01	0.002
Δ*codY*	34.5	±0.345	73.39	3.85	0.14	0.064
(p)ppGpp^0 *gmk* ^{Q110R}	33.3	±0.333	61.04	0.48	0.10	0.026
guaB ^{Down}	37.1	±0.371	73.61	3.54	1.85	0.293
Δ*guaA*	835.12	±56.262	81.72	11.86	17.37	6.993
Δ*purF*	495.11	±35.55	76.58	5.26	11.74	4.028
Δ*purB*	3013.68	±264.06	76.32	7.11	20.51	5.757
Δ*purL*	577.62	±48.47	81.08	6.91	17.90	4.055
Δ*pyk*	55.8	±0.558	77.52	2.36	0.01	0.007
Δ*ctaA*	38.6	±0.386	63.22	1.16	0.01	0.003
WT ΔΦ	29.4	±0.294	69.19	2.73	0.10	0.059
(p)ppGpp^0 ΔΦ	29	±0.29	64.17	0.36	0.01	0.003

[a]Exponential growth rates were measured from growth in the same growth media as the antibiotic killing assays.

[b]Tolerance was determined from the killing phase of the survival curve as estimated MDK_{99} (minimal duration of killing of 99% of population) from Fig 2b-c and supplemental data figure 4.

[c]Persistence was determined from the fraction of survivors in the antibiotic refractory phase (5h) in the survival curves from Fig 2b-c and supplemental data figure 4.

Supplemental Data Table 8. **List of Tnseq gene disruptions after Vancomycin treatment with > 30k reads post-treatment and false discovery rate (FDR) < 0.01. Genes highlighted are involved in GTP biosynthesis.**

Van treatment vs untreated				
**logFC**	**fdr**	**hitCount**	**Name**	**product**
9.81551	1.95E-12	48666	*pyrE*	phosphoribosyltransferase
9.66084	2.34E-12	94898	*purK*	5-(carboxyamino)imidazole ribonucleotide synthase
9.30648	6.42E-12	227624	*mntR*	transcriptional regulator MntR
9.15585	1.21E-11	37640	*pyrK*	dihydroorotate dehydrogenase B
8.82884	4.34E-11	30914	*pyrC*	dihydroorotase
8.5703	9.66E-11	108567	*purA*	adenylosuccinate synthase
8.48135	1.11E-10	206494	*carB*	synthase pyrimidine-specific large chain
8.43738	1.23E-10	201911	*purM*	phosphoribosylformylglycinamidine cyclo-ligase
8.40323	1.40E-10	54786	*pyrB*	carbamoyltransferase
8.32947	1.67E-10	117995	*purC*	ole-succinocarboxamide synthase
8.23874	2.12E-10	240473	*purL*	phosphoribosylformylglycinamidine synthase
8.22247	2.12E-10	210940	*purD*	phosphoribosylamine--glycine ligase
7.76277	1.34E-09	84146	*purS*	hypothetical protein
7.39986	5.57E-09	41716	*purF*	amidophosphoribosyltransferase
11.8434	9.68E-09	367	*guaA*	GMP synthase
7.11908	1.47E-08	77637	*purN*	phosphoribosylglycinamide formyltransferase
7.01813	2.03E-08	138384	*purH*	bifunctional purine biosynthesis protein PurH
7.03071	2.03E-08	36388	*yeaB*	transporter
6.82725	4.47E-08	50042	*pyrAA*	synthase pyrimidine-specific small chain
6.45502	1.82E-07	506414	*efeU*	ferrous iron permease EfeU
6.15153	5.97E-07	224169	*efeM*	iron uptake system component EfeM
5.61549	4.51E-06	182075	*efeN*	deferrochelatase/peroxidase EfeN
5.61297	4.51E-06	153875	*purQ*	phosphoribosylformylglycinamidine synthase
4.55192	0.00022	33941	*ccpA*	catabolite control protein A
4.02673	0.001366	119500	*nifS*	cysteine desulfurase NifS
3.87275	0.00211	96854	*nadA*	quinolinate synthase A
3.76742	0.002937	83500	*nadC*	nicotinate-nucleotide pyrophosphorylase
3.7476	0.003115	120935	*nadB*	L-aspartate oxidase

Supplemental Data Table 9. **List of Tnseq gene disruptions after Ciprofloxacin treatment with > 30k reads post-treatment and false discovery rate (FDR) < 0.01. Genes highlighted are involved in GTP biosynthesis.**

Cip treatment vs untreated				
**logFC**	**fdr**	**hitCount**	**Name**	**product**
17.5055	1.3E-17	16107	*guaA*	GMP synthase
12.0533	4.12E-16	65650	*purB*	adenylosuccinate lyase
11.3777	2.33E-15	248283	*purK*	5-(carboxyamino)imidazole ribonucleotide synthase
10.7585	1.48E-14	802982	*purM*	phosphoribosylformylglycinamidine cyclo-ligase
10.753	1.48E-14	392279	*purA*	adenylosuccinate synthase
10.5099	3.40E-14	819512	*purD*	phosphoribosylamine--glycine ligase
10.0511	2.05E-13	672137	*purL*	phosphoribosylformylglycinamidine synthase
9.7303	7.27E-13	247959	*purC*	phosphoribosylaminoimidazole-succinocarboxamide synthase
9.58473	1.15E-12	652420	*purH*	bifunctional purine biosynthesis protein PurH
9.37591	2.21E-12	204862	*purS*	hypothetical protein
9.33953	2.42E-12	143487	*yeaB*	transporter
9.20974	3.53E-12	116402	*purF*	amidophosphoribosyltransferase
9.15634	3.98E-12	253611	*purN*	phosphoribosylglycinamide formyltransferase
8.91895	9.97E-12	138479	*mntR*	transcriptional regulator MntR
7.47286	2.52E-09	60609	*yorE*	hypothetical protein
7.41833	2.93E-09	428008	*purQ*	phosphoribosylformylglycinamidine synthase
6.71729	3.82E-08	33949	*pyrR*	bifunctional protein PyrR
6.66331	4.34E-08	249102	*yqjI*	6-phosphogluconate dehydrogenase
4.96425	2.12E-05	56995	*sodA*	superoxide dismutase
4.42502	0.00015	113027	*nadA*	quinolinate synthase A
4.31627	0.00023	97213	*nadC*	nicotinate-nucleotide pyrophosphorylase
4.17728	0.00037	129632	*nadB*	L-aspartate oxidase
3.89781	0.00095	72705	*ydbI*	hypothetical protein
3.55122	0.00304	78220	*yecA*	amino acid permease
