# Supplementary material for: A shared alarmone–GTP switch controls persister formation in bacteria
Source: Nat Microbiol. 2025 May 15;10(7):1617–29. doi: 10.1038/s41564-025-02015-6 (PMC12221987; doi:10.1038/s41564-025-02015-6)
Supplement: Supplementary file 2 — Reporting Summary [file 41564_2025_2015_MOESM2_ESM.pdf]

nature portfolio

Corresponding author(s): Jue D Wang

Last updated by author(s): Apr 7, 2025

Reporting Summary

Nature Portfolio wishes to improve the reproducibility of the work that we publish. This form provides structure for consistency and transparency in reporting. For further information on Nature Portfolio policies, see our Editorial Policies and the Editorial Policy Checklist.

Statistics

For all statistical analyses, confirm that the following items are present in the figure legend, table legend, main text, or Methods section.

n/a	Confirmed	
☐	☒	The exact sample size (*n*) for each experimental group/condition, given as a discrete number and unit of measurement
☐	☒	A statement on whether measurements were taken from distinct samples or whether the same sample was measured repeatedly
☐	☒	The statistical test(s) used AND whether they are one- or two-sided<br>*Only common tests should be described solely by name; describe more complex techniques in the Methods section.*
☒	☐	A description of all covariates tested
☒	☐	A description of any assumptions or corrections, such as tests of normality and adjustment for multiple comparisons
☐	☒	A full description of the statistical parameters including central tendency (e.g. means) or other basic estimates (e.g. regression coefficient) AND variation (e.g. standard deviation) or associated estimates of uncertainty (e.g. confidence intervals)
☐	☒	For null hypothesis testing, the test statistic (e.g. *F*, *t*, *r*) with confidence intervals, effect sizes, degrees of freedom and *P* value noted<br>*Give P values as exact values whenever suitable.*
☒	☐	For Bayesian analysis, information on the choice of priors and Markov chain Monte Carlo settings
☒	☐	For hierarchical and complex designs, identification of the appropriate level for tests and full reporting of outcomes
☐	☒	Estimates of effect sizes (e.g. Cohen's *d*, Pearson's *r*), indicating how they were calculated

Our web collection on statistics for biologists contains articles on many of the points above.

Software and code

Policy information about availability of computer code

Data collection	Metamorph Advanced (v 7.8.3.0) and Olympus cellSense Dimension 2.2 for microscopy data collection. BD FACSDiva (v 8.0.2) for flow cytometry data collection.
Data analysis	Graphpad Prism (version 7.0) for statistical analysis. Maven for metabolome data analysis. Metamorph Advanced (v 7.8.3.0) for imaging data analysis. FlowJo X for flow cytometry data analysis. ImageQuant 5.0 for thin layer chromatography image analysis. A custom python script and R for analysis of sequencing data.

For manuscripts utilizing custom algorithms or software that are central to the research but not yet described in published literature, software must be made available to editors and reviewers. We strongly encourage code deposition in a community repository (e.g. GitHub). See the Nature Portfolio guidelines for submitting code & software for further information.

Data

Policy information about availability of data

All manuscripts must include a data availability statement. This statement should provide the following information, where applicable:

- Accession codes, unique identifiers, or web links for publicly available datasets
- A description of any restrictions on data availability
- For clinical datasets or third party data, please ensure that the statement adheres to our policy

Data generated in this study are included in the figures and supplemental data as much as possible. The sequencing data used for Tn-seq analysis is available at SRA

under BioProject Accession Number PRJNA1229644. Any other relevant data that supports the findings of this study are available from the corresponding author upon request.

Research involving human participants, their data, or biological material

Policy information about studies with human participants or human data. See also policy information about sex, gender (identity/presentation), and sexual orientation and race, ethnicity and racism.

Reporting on sex and gender	not applicable
Reporting on race, ethnicity, or other socially relevant groupings	not applicable
Population characteristics	not applicable
Recruitment	not applicable
Ethics oversight	not applicable

Note that full information on the approval of the study protocol must also be provided in the manuscript.

Field-specific reporting

Please select the one below that is the best fit for your research. If you are not sure, read the appropriate sections before making your selection.

☒ Life sciences ☐ Behavioural & social sciences ☐ Ecological, evolutionary & environmental sciences

For a reference copy of the document with all sections, see nature.com/documents/nr-reporting-summary-flat.pdf

Life sciences study design

All studies must disclose on these points even when the disclosure is negative.

Sample size	No sample-size calculation was performed. Sample sizes were chosen to show statistical significance while considering material availability. Our sample sizes were similar to previous publications (PMID: 27572640, PMID: 27398229)
Data exclusions	No data was excluded.
Replication	Experiments were repeated at least three times and were successful.
Randomization	Experiments were done using isogenic Bacillus subtilis strains. The samples are not randomized to control for inoculation densities and culture properties which is critical for measurement of antibiotic survival phenotypes.
Blinding	No blinding during data acquisitions can be done since comparisons were made before and after antibiotic treatment over time. However, rigorous data analysis procedures were implemented to avoid bias and any analysis methods were applied the same way across data sets.

Reporting for specific materials, systems and methods

We require information from authors about some types of materials, experimental systems and methods used in many studies. Here, indicate whether each material, system or method listed is relevant to your study. If you are not sure if a list item applies to your research, read the appropriate section before selecting a response.

Materials & experimental systems

n/a	Involved in the study	
☒	☐	Antibodies
☒	☐	Eukaryotic cell lines
☒	☐	Palaeontology and archaeology
☒	☐	Animals and other organisms
☒	☐	Clinical data
☒	☐	Dual use research of concern
☒	☐	Plants

Methods

n/a	Involved in the study	
☒	☐	ChIP-seq
☐	☒	Flow cytometry
☒	☐	MRI-based neuroimaging

Plants

Seed stocks: not applicable

Novel plant genotypes: not applicable

Authentication: not applicable

Flow Cytometry

Plots

Confirm that:

☒ The axis labels state the marker and fluorochrome used (e.g. CD4-FITC).

☒ The axis scales are clearly visible. Include numbers along axes only for bottom left plot of group (a 'group' is an analysis of identical markers).

☒ All plots are contour plots with outliers or pseudocolor plots.

☒ A numerical value for number of cells or percentage (with statistics) is provided.

Methodology

Sample preparation: Bacterial cells were grown in synthetic media and fixed with 0.4% paraformaldehyde for 15 min at room temperature, washed 3x with 1x phosphate buffered saline (PBS), and kept at 4°C until analysis on the same day. Fixation was verified by viability plating and microscopy. No fixation was performed for samples subjected to cell sorting.

Instrument: BD LSRFortessa flow cytometer (BD Biosciences) for flow cytometry and BD FACSAria cell sorter (BD Biosciences) for cell sorting

Software: BD FACSDiva v8.0.2 (BD Biosciences) was used for operation of the instruments

Cell population abundance: At least two million events were counted for each sample. For FACS sorting, approximately five million cells were sorted so that at least 5,000 cells were obtained from the rarest gate for each sample.

Gating strategy: Cell populations were pre-gated using both forward and side scatter (FSC and SSC). An example gating strategy is included in Supplemental Data Figure 14a. Cell recovery rate was estimated to be > 90% based on viability counting on LB plates. Cells were subsequently gated based on the PlowGTP fluorescence reporter and sorted by high fluorescence cutoff. An example for determining this cutoff is included in Fig 3b.

☒ Tick this box to confirm that a figure exemplifying the gating strategy is provided in the Supplementary Information.
